# Role of β-adrenergic signaling in masseter muscle

**DOI:** 10.1371/journal.pone.0215539

**Published:** 2019-04-15

**Authors:** Aiko Ito, Yoshiki Ohnuki, Kenji Suita, Misao Ishikawa, Yasumasa Mototani, Kouichi Shiozawa, Naoya Kawamura, Yuka Yagisawa, Megumi Nariyama, Daisuke Umeki, Yoshiki Nakamura, Satoshi Okumura

**Affiliations:** 1 Department of Physiology, Tsurumi University School of Dental Medicine, Yokohama, Japan; 2 Department of Orthodontics, Tsurumi University School of Dental Medicine, Yokohama, Japan; 3 Department of Oral Anatomy, Tsurumi University School of Dental Medicine, Yokohama, Japan; 4 Department of Periodontology, Tsurumi University School of Dental Medicine, Yokohama, Japan; 5 Department of Pediatric Dentistry, Tsurumi University School of Dental Medicine, Yokohama, Japan; Rush University Medical Center, UNITED STATES

## Abstract

In skeletal muscle, the major isoform of β-adrenergic receptor (β-AR) is β_2_-AR and the minor isoform is β_1_-AR, which is opposite to the situation in cardiac muscle. Despite extensive studies in cardiac muscle, the physiological roles of the β-AR subtypes in skeletal muscle are not fully understood. Therefore, in this work, we compared the effects of chronic β_1_- or β_2_-AR activation with a specific β_1_-AR agonist, dobutamine (DOB), or a specific β_2_-AR agonist, clenbuterol (CB), on masseter and cardiac muscles in mice. In cardiac muscle, chronic β_1_-AR stimulation induced cardiac hypertrophy, fibrosis and myocyte apoptosis, whereas chronic β_2_-AR stimulation induced cardiac hypertrophy without histological abnormalities. In masseter muscle, however, chronic β_1_-AR stimulation did not induce muscle hypertrophy, but did induce fibrosis and apoptosis concomitantly with increased levels of p44/42 MAPK (ERK1/2) (Thr-202/Tyr-204), calmodulin kinase II (Thr-286) and mammalian target of rapamycin (mTOR) (Ser-2481) phosphorylation. On the other hand, chronic β_2_-AR stimulation in masseter muscle induced muscle hypertrophy without histological abnormalities, as in the case of cardiac muscle, concomitantly with phosphorylation of Akt (Ser-473) and mTOR (Ser-2448) and increased expression of microtubule-associated protein light chain 3-II, an autophagosome marker. These results suggest that the β_1_-AR pathway is deleterious and the β_2_-AR is protective in masseter muscle. These data should be helpful in developing pharmacological approaches for the treatment of skeletal muscle wasting and weakness.

## Introduction

Adrenergic receptors (ARs) belong to the guanine nucleotide-binding G-protein-coupled receptor (GPCR) family. Among them, β_2_-AR is the most abundant form in skeletal muscle, while β_1_-AR accounts for less than 10% of ARs, and there are small populations of α_1_-AR and β_3_-AR [1]. In contrast, the predominant receptor subtype expressed in the heart is β_1_-AR, with approximately 20% of β_2_-AR [2].

The physiological roles of β_1_- and β_2_-AR in the heart have been extensively investigated using both pharmacological [3,4] and gene-targeting approaches [5]. β_1_-AR-mediated cAMP signaling is involved in catecholamine-induced cardiac myocyte hypertrophy in vitro [4] and in vivo [6]. Furthermore, stimulation of β_1_-AR triggers cardiac myocyte apoptosis via a cyclic AMP (cAMP)-dependent mechanism [5,7] and maladaptive cardiac remodeling in vivo [8]. In contrast to the well-established deleterious cardiac effects of β_1_-AR, β_2_-AR stimulation on the heart delivers cardiac hypertrophy with an antiapoptotic effect through the Giα-Gβγ-phosphoinositol 3-kinase (PI3K)-Akt cell survival pathway [9,10].

Previous studies of the physiological significance of β-AR signaling in skeletal muscle have focused on the role of β_2_-AR stimulation, using selective β_2_-AR agonists. Chronic stimulation of β_2_-AR with a selective β_2_-AR agonist, clenbuterol (CB), induced skeletal muscle hypertrophy via activation of Akt/mechanistc target of rapamycin (mTOR) signaling [11,12], and while histological analysis revealed no abnormality (such as fibrosis), the contractility might be increased, as in the case of cardiac muscle, even though resistance to fatigue was reported to be decreased [11,13–15]. In contrast, the physiological significance of β_1_-AR signaling in skeletal muscle remains less extensively investigated, probably due to the low expression level (less than 10% of ARs). But, the physiological importance of β-AR subtypes may depend upon their downstream signaling rather than their expression levels [12].

mTOR regulates cell growth and metabolism via two structurally and functionally distinct mTOR-containing protein complexes, mTORC1 and mTORC2, in response to environmental cues [16]. It was recently reported that mTOR phosphorylation at serine 2448 (mTORC1) is regulated by phosphoinositide 3-kinase (PI3)-Akt signaling and mTOR phosphorylation at serine 2481 (mTORC2) is regulated through the cAMP-PKA pathway in skeletal muscle [11,17]. However, the effects of β-AR subtype-specific stimulation on mTORC1 and mTORC2 remain largely unknown.

Various pathophysiological factors are involved in the progression of muscle dysfunction, including Ca^2+^ homeostasis in skeletal and cardiac muscles [18,19]. The function of the sarcoendoplasmic reticulum (SR) calcium transport ATPase (SERCA2a), a major player in Ca^2+^ homeostasis, is modulated by phospholamban (PLN) in skeletal and cardiac muscles [20]. PLN is a low-molecular weight phosphoprotein in both cardiac and skeletal SR, and dephosphorylated PLN is an inhibitor of SERCA-mediated transport of Ca^2+^. Thus, PLN plays an important role for the regulation of Ca^2+^ homeostasis in skeletal and cardiac muscle [20]. Following β-AR activation, and thus production of cAMP by adenylyl cyclase, protein kinase A (PKA) phosphorylates PLN on serine 16 and CaMKII phosphorylates PLN on threonine 17 [21]. Importantly, increased PLN phosphorylation or overexpression of PLN exaggerates cardiac function, leading to increased cardiac fibrosis and apoptosis in mice, as established by us and other groups [22–24]. It was recently reported that progressive fibrosis and muscle weakness are induced in mice overexpressing PLN in skeletal muscle [25], but the role of the overexpressed PLN in skeletal muscle is still not clearly understood.

We hypothesized that β_1_-AR-mediated signaling and its downstream molecules might play an important role in the development of skeletal muscle dysfunction, and to test this idea, we examined and compared the effects of selective stimulation of β_1_- or β_2_-AR in masseter muscle and cardiac muscle by chronic infusion of a selective β_1_-AR agonist, dobutamine (DOB), or a selective β_2_-AR agonist, CB, for 1 week in mice.

## Materials and methods

### Mice and experimental protocols

All experiments were performed on male 12-week-old C57BL/6 mice obtained from CLEA Japan (Tokyo, Japan). Mice were group-housed at 23°C in under a 12–12 light/dark cycle with lights on at 8:00 AM. Food and water were available ad libitum. This study was approved by the Animal Care and Use Committees of Tsurumi University.

**Fig 1:**
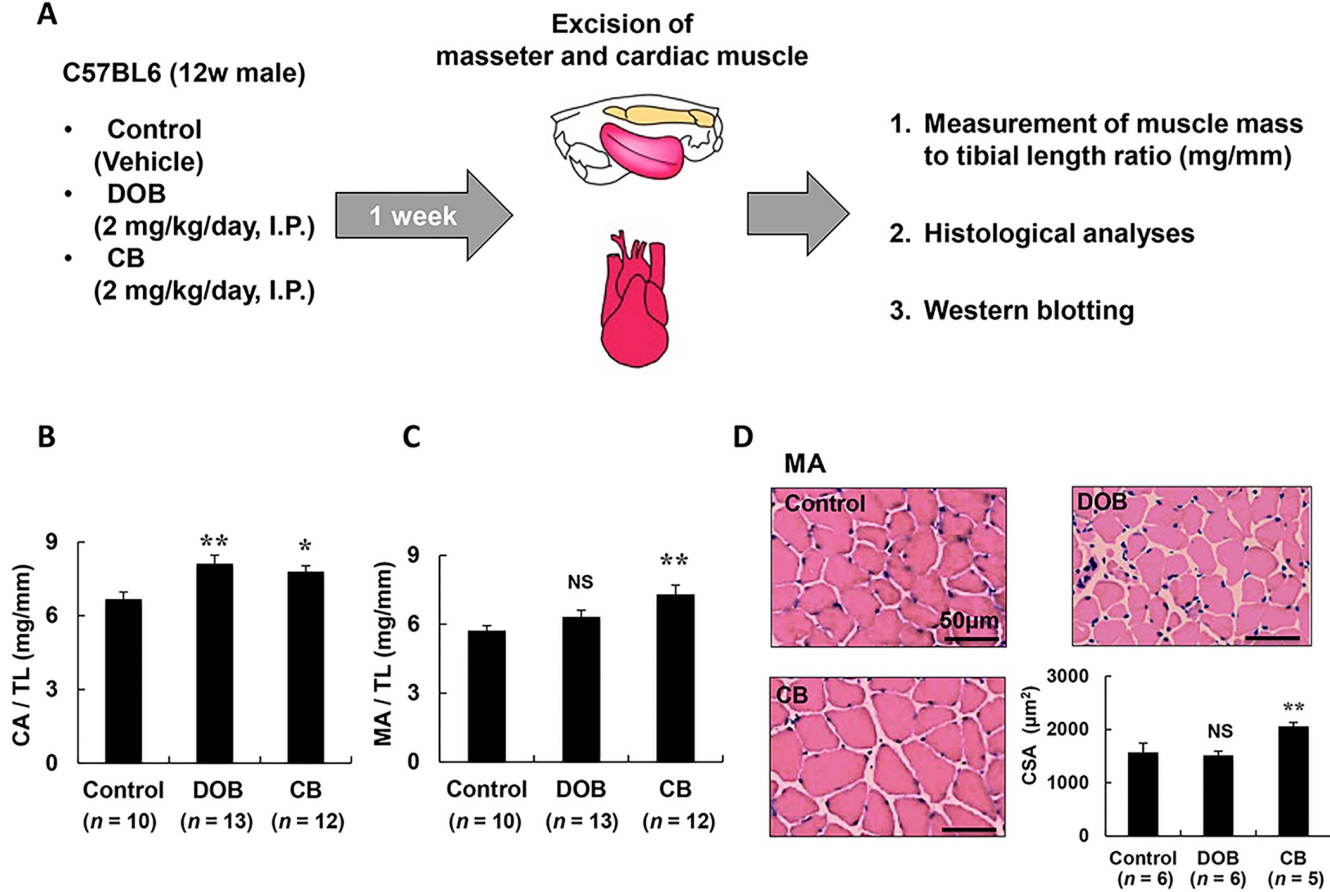
Experimental procedure, daily consumption of food and water, and body weight. **(A)** Dobutamine (DOB) and clenbuterol (CB) was administered once daily for 1 week via intraperitoneal injection (I.P.) at a dose of 2 mg/kg, dissolved in saline. Age-matched control mice (Control) received an identical volume of saline only. **(B)** In cardiac muscle (CA), the muscle mass per tibial length ratio was significantly increased in the DOB group (**P < 0.01 vs. Control), as well as the CB group (*P < 0.05 vs. Control). **(C)** In masseter muscle (MA), the muscle mass per tibial length ratio was significantly increased in the CB group (**P < 0.01 vs. Control), but not in the DOB group **(D)** Typical cross-sections of HE staining of masseter muscle (MA) in the three groups (Control (*upper left*), DOB (*upper right*) and CB (*lower left*)). CSA was significantly increased in the CB group (**P < 0.01 vs. Control), but not in the DOB group (*lower right*). Scale bars: 50 μm.

DOB (Sigma, St. Louis, MO, USA) and CB (Sigma) were each dissolved in saline to prepare a 0.6 mg/ml stock solution and the appropriate volume of this solution to provide the desired dose (2mg/kg) was added to 0.2 ml of saline to prepare the solution for intraperitoneal (i.p.) injection [11,26]. DOB or CB was administered i.p. once daily for 1 week, while control mice received an identical volume of saline only **(Fig 1A)**. β_1_-AR agonist DOB and β_2_-AR agonist CB were administered in equal volume doses (2mg/kg) to examine the subtype-specific effects of β-AR in mice, in line with previous studies [11,12,27]. Body weight, food intake, and water intake were monitored for all animals throughout the 1-week experimental period. The dose of CB used in this study has been reported to increase skeletal mass efficiently without affecting body weight [28]. After the completion of each treatment, mice were anesthetized with isoflurane and the heart, left and right masseter muscle were excised, rinsed thoroughly in phosphate-buffered saline (PBS) to eliminate circulating blood in tissue, blotted on filter paper, weighted, frozen, and stored at -80°C.

The muscle mass (mg) and the ratio of muscle mass to tibial length (mm) were used as indexes of muscle growth. After tissue extraction, the mice were killed by cervical dislocation [29]. The protocol is summarized in **Fig 1A**.

### Cross-sectional area of muscle fibers

The left masseter muscle, excised as a whole **(Fig 1A)**, was embedded in Tissue-Tek OCT compound (Sakura Finetec, Torrance, CA, USA) in a slightly stretched state so as to maintain a length close to the resting length (L0), and stored at -80°C until sectioning, as reported [30]. Cross sections (10μm) were cut from the middle portion of the left masseter muscle with a cryostat (CM1900, Leica Microsystems, Nussloch, Germany) at -20°C. The section were air-dried and fixed with 4% paraformaldehyde in 0.1 M PBS (pH 7.5). The sections were then stained with hematoxylin and eosin (HE) and observed under a light microscope (BX61, Olympus Co., Tokyo, Japan). Micrographs were taken with a digital camera (DP-72, Olympus Co.) connected to a personal computer. The cross-sectional size of muscle fibers was evaluated by measuring the cross-sectional area (CSA) [31,32]. The CSA of 100 muscle fibers in the superficial portion was measured with image analysis software (Image J 1.45) and averaged to obtain the mean values in each mouse.

### Evaluation of fibrosis

Among several quantitative methods that are available to determine interstitial fibrotic regions [22,33,34], we employed Masson-trichrome staining using the Accustatin Trichrome Stain Kit (#HT15-1KT; Sigma) in accordance with the manufacturer’s protocol, as described previously [22,35]. Interstitial fibrotic regions were quantified using image analysis software (Image J 1.45) to evaluate the percentage of blue area in the Masson-trichrome section [22].

### Evaluation of apoptosis

Apoptosis was determined by terminal deoxyribonucleotidyl transferase (TdT)-mediated biotin-16-deoxyuridine triphosphate (dUTP) nick-end labeling (TUNEL) staining using the Apoptosis *in situ* Detection Kit (#293–71501; Wako, Osaka, Japan). TUNEL-positive nuclei per field of view were manually counted in six sections of three groups (Control, DOB and CB) over a microscopic field of 20 x, averaged and expressed as the ratio of TUNEL-positive nuclei (%) [22,32]. Limiting the counting of total nuclei and TUNEL-positive nuclei to areas with a true cross section of myocytes made it possible to selectively count only those nuclei that were clearly located within myocytes. For some samples, TUNEL staining was performed using the In Situ Cell Death Detection Kit, Fluorescein (#11684795910; Sigma) with simultaneous immunostaining of dystrophin for muscle membrane identification and Hoechst staining for visualization of nuclei; the results confirmed that apoptotic cells identified and counted as TUNEL-positive nuclei by light microscopy were located inside the myofibers.

### Evaluation of the number of microvessels per masseter myocyte

Frozen cross sections of the masseter muscle were immunohistochemically double-stained with antibodies against dystrophin (#D8168, Sigma) and CD31 (#ab28365, Abcam, Cambridge, UK). The number of microvessels per masseter myocyte was calculated as described previously [22,36].

### Skinned muscle strand preparation and measurements of isometric force and ATPase activity

Muscle strands (5 mm long, 2 mm wide and 2 mm thick) were excised from the masseter muscle and skinned muscle strands were prepared as reported [37,38]. Isometric force and ATPase activity were measured in the relaxing solution (pCa 8.0) and the activating solutions with the various Ca^2+^ concentrations (pCa 6.1, 5.8, 5.5, 4.6) as described previously [37,38]. Results were analyzed and compared using Microsoft Excel 2010 (Microsoft Corporation, Redmond, WA, USA) and Igor Pro 3.15 (HULINKS Inc., Tokyo, Japan).

### Western blotting

The excised right masseter muscle was homogenized in a Polytron (Kinematica AG, Lucerne, Switzerland) in ice-cold RIPA buffer (Thermo Fisher Scientific, Waltham, MA, USA: 25mM Tris-HCl (pH 7.6), 150mM NaCl, 1% NP-40, 1% sodium deoxycholate, 0.1% SDS) without addition of inhibitors [39], and the homogenate was centrifuged at 13,000 x *g* for 10 min at 4°C. The supernatant was collected and the protein concentration was measured using a DC protein assay kit (Bio-Rad, Hercules, CA, USA). Equal amounts of protein (5 μg) were subjected to 12.5% SDS-polyacrylamide gel electrophoresis and blotted onto 0.2 mm PVDF membrane (Millipore, Billerica, MA, USA).

Western blotting was conducted with commercially available antibodies [22,32,40,41]. The primary antibodies against Akt (#9272), phospho-Akt (Ser-473, #9721), CaMKII (#3362), phospho-CaMKII (Thr-286, #3361), p44/42 MAPK (ERK1/2) (#4695), phospho-ERK1/2 (Thr-202/Tyr-204, #4370), BAX (#2772), microtubule-associated protein light chain 3 (LC3; #12741), mTOR (#2972), phospho-mTOR (Ser-2448, #5536; Ser-2481, #2974) and β_1_-AR (#12271) were purchased from Cell Signaling Technology (Boston, MA, USA), the primary antibodies against GAPDH (sc-25778) and β_2_-AR (sc-569) were purchased from Santa Cruz Biotechnology (Santa Cruz, CA, USA), the primary antibody against μ-calpain was purchased from Sigma, and the primary antibodies against phospholamban (PLN) (#A010-14) and phospho-PLN (Ser-16, #A010-12; Thr-17, #A010-13) were purchased from Badrilla (Leeds, UK). Horseradish peroxide-conjugated anti-rabbit or anti-mouse IgG (#NA934; GB Healthcare, Piscataway, NJ, USA) was used as a secondary antibody. The primary and secondary antibodies were diluted in Tris-buffered saline (pH 7.6) with 0.1% Tween 20 and 5% bovine serum albumin. The blots were visualized with enhanced chemiluminescence solution (ECL Prime Western Blotting Detection Reagent, GE Healthcare) and scanned with a densitometer (LAS-1000, Fuji Photo Film, Tokyo, Japan).

### Cell culture and evaluation of apoptosis

Murine myoblast C2C12 cells were maintained as described previously [42]. In brief, cells were cultured in Dulbecco’s modified Eagle’s medium (DMEM; Fisher Scientific, Loughborough, UK) supplemented with 10% fetal bovine serum (FBS; Fisher Scientific, Loughborough, UK), 100 U/ml penicillin (Fisher Scientific, Loughborough, UK), and 100 U/ml streptomycin (Fisher Scientific) at 37°C in 5% CO_2_ and 95% air at 100% humidity. For the induction of differentiation, confluent cells were cultured in DMEM supplemented with 2% horse serum (Fisher Scientific; differentiation medium) and the antibiotics mentioned above for 4 days.

To examine subtype-specific apoptosis, DOB (β_1_ agonist) or CB (β_2_ agonist) was diluted in differentiation medium and added to the differentiated C2C12 myotubes at 10^−4^ M. The myotubes were further cultured for 24 hr and stained with propidium iodide (PI; DOJINDO, Kumamoto, Japan) together with immunohistochemistry with an anti-myosin heavy chain antibody for detection of myosin and Hoechst staining for detection of nuclei [43]. We performed preliminary experiments in the concentration range of 10^−4^–10^−6^ M, with reference to the previous study, and determined that 10^−4^ M was the optimal concentration for both DOB and CB [44].

### Statistical analysis

All data are reported as mean ± SEM. Comparison of data was performed using ANOVA followed by Tukey’s post test. Differences were considered significant when *P* < 0.05.

## Results

### Effects of DOB and CB on body weight and consumption of food and water

We monitored the daily consumption of pellet food **(S1A Fig)** and water **(S1B Fig)**, and confirmed that there were no significant differences among the three groups (food: DOB (*n* = 13): 4.0 ± 0.2 g, CB (*n* = 12): 3.0 ± 0.4 g, Control (*n* = 10): 3.6 ± 0.2 g, *P* = NS (not significant) for DOB or CB vs. Control; water: DOB (*n* = 13): 7.3 ± 0.3 mL, CB (*n* = 12): 5.6 ± 0.7 mL, Control (*n* = 10): 6.3 ± 0.1 mL, *P* = NS for DOB or CB vs. Control). Body weight also showed no difference among the three groups before (Control (26 ± 0.5 mg), DOB (25 ± 0.6 mg) and CB (25 ± 0.7 mg)) and after the 1-week treatment period (Control (26 ± 0.5 mg), DOB (26 ± 0.6 mg) and CB (27 ± 0.6 mg)) [45] (*n* = 6 in each group, *P* = NS for DOB or CB vs. Control (0 day)) **(S1C Fig)**.

### Masseter muscle mass was significantly increased by CB but not by DOB

We next evaluated cardiac and masseter muscle hypertrophy in terms of muscle mass **(S1D Fig and S1E Fig)** and the muscle mass per tibia length ratio (mg/mm) **(Fig 1B and 1C)**. In cardiac muscle, cardiac muscle mass and the cardiac muscle mass to tibial length ratio were significantly increased in the DOB group (cardiac muscle mass: Control (*n* = 10) vs. DOB (*n* = 10): 127 ± 3.3 vs. 172 ± 9.6 mg, *P* < 0 .01; cardiac muscle mass/tibial lengh ratio: Control (*n* = 10) vs. DOB (*n* = 13): 6.7 ± 0.3 vs. 8.1 ± 0.4 mg/mm, *P* < 0.01), as well as in the CB group (cardiac muscle mass: Control (*n* = 10) vs. DOB (*n* = 10): 127 ± 3.3 vs. 162 ± 10.5 mg, *P* < 0 .01; cardiac muscle mass/tibial length ratio: Control (*n* = 10) vs. CB (*n* = 12): 6.7 ± 0.3 vs. 7.8 ± 0.2, *P* < 0.05) **(S1D Fig and S1B Fig)**. There was no significant difference between the two agonists, in agreement with reported findings [46]. However, in masseter muscle, the ratio was significantly increased in the CB group (masseter muscle mass: Control (*n* = 10) vs. CB (*n* = 10): 114 ± 2.9 vs. 168 ± 4.0 mg, *P* < 0 .01; masseter muscle mass/tibial length ratio: Control (*n* = 10) vs. CB (*n* = 12): 5.7 ± 0.2 vs. 7.3 ± 0.4 mg/mm, *P* < 0.05), but not in the DOB group (masseter muscle mass: Control (*n* = 10) vs. DOB (*n* = 10): 114 ± 2.9 mg vs. 115 ± 2.6 mg, *P* = NS; masseter muscle mass/tibial length ratio: Control (*n* = 10) vs. DOB (*n* = 13): 5.7 ± 0.2 vs. 6.3 ± 0.3 mg/mm, *P* = NS) **(S1E Fig and S1C Fig)**.

Next, to confirm the difference between the effects of DOB and CB in masseter muscle, we performed HE staining of masseter muscle and measured the fiber CSA **(Fig 1D)**. The CSA was significantly increased in the CB group (Control (*n* = 6) vs. CB (*n* = 5): 1525 ± 149 vs. 2060 ± 72 μm^2^, *P* < 0.01), but not in the DOB group (Control (*n* = 6) vs. DOB (*n* = 6): 1525 ± 149 vs. 1494 ± 69 μm^2^, *P* = NS) **(Fig 1D)**.

These data indicate that masseter muscle hypertrophy was mediated through the activation of β_2_-AR, but not β_1_-AR, whereas cardiac hypertrophy was mediated through the activation of both β_2_-AR and β_1_-AR.

### Fibrosis was similarly and significantly increased by DOB in both cardiac and masseter muscles

**Fig 2:**
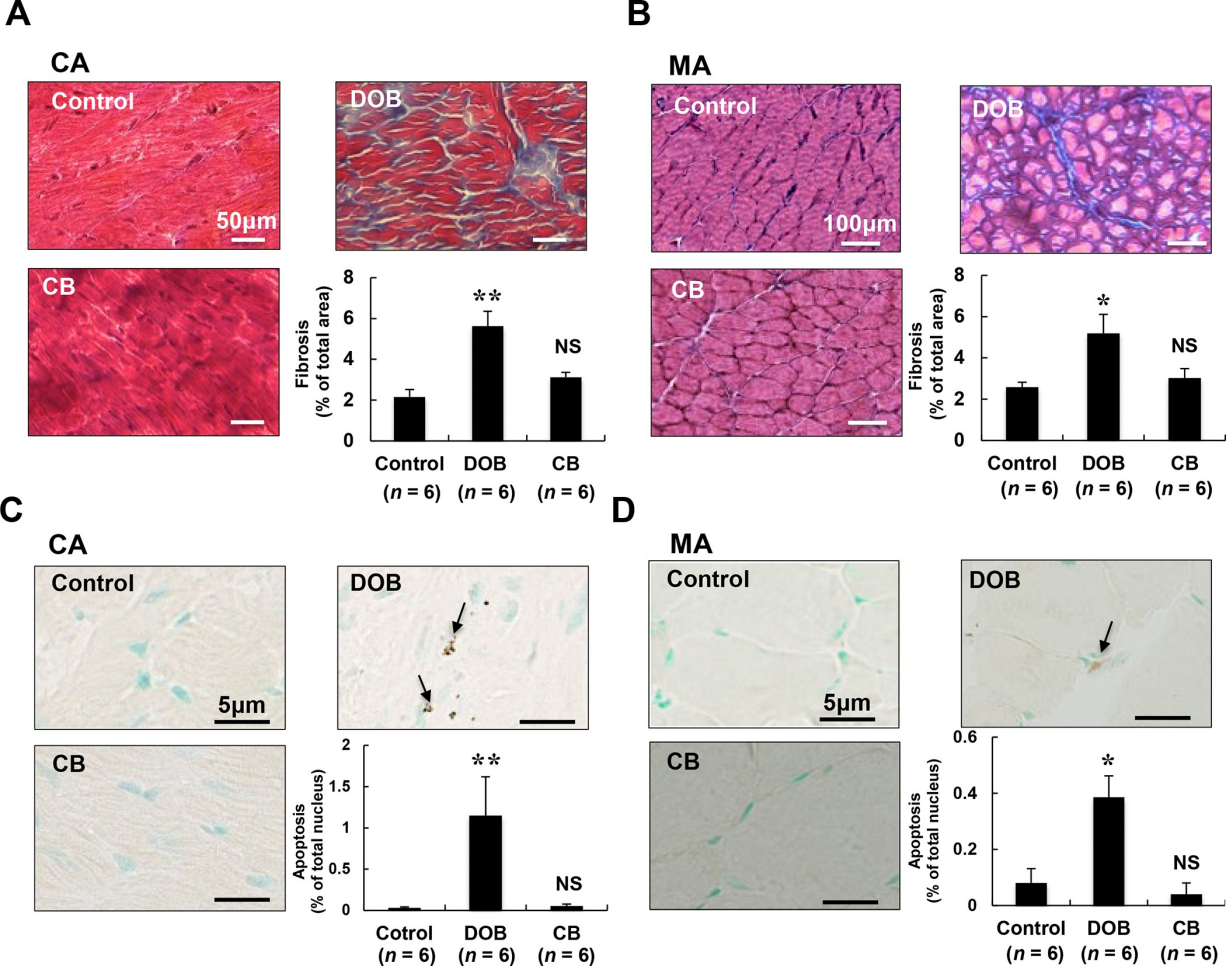
Effects of DOB and CB on fibrosis and apoptosis in cardiac and masseter muscles. **(A-B)** Representative images of Masson-trichrome-stained sections of cardiac muscle **(A)** and masseter muscle **(B)** in the Control (*upper left*), DOB (*upper right*) and CB (*lower left*) groups. The area of fibrosis was significantly increased in both the DOB-treated cardiac **(A)** and masseter **(B)** muscle (*P < 0.05, **P < 0.01 vs. Control), but not in the CB-treated cardiac **(A)** or masseter **(B)** muscle (*lower right*). Scale bars: 50 μm **(A)** and 100 μm **(B)**. **(C-D)** Representative images of TUNEL-stained sections of cardiac muscle **(C)** and masseter muscle **(D)** in the Control (*upper left*), DOB (*upper right*) and CB (*lower left*) groups. TUNEL-positive nuclei (black arrows) were counted in cardiac muscle **(C)** and masseter muscle **(D)** after 1 week of DOB or CB infusion and expressed as percentage of total myocytes (*lower right*). The number of TUNEL-positive nuclei was significantly increased in both the DOB-treated cardiac muscle (**P < 0.01 vs. Control) and masseter muscle (*P < 0.05 vs. Control), but not in the CB-treated cardiac or masseter muscle. Scale bars: 5 μm **(C)** and **(D)**. CA; cardiac muscle, MA; masseter muscle.

We next examined the effects of chronic DOB or CB infusion on fibrosis in cardiac and masseter muscles by means of Masson-trichrome staining **(Fig 2A and 2B)**. DOB significantly increased the area of fibrosis in cardiac **(Fig 2A)** and masseter muscles **(Fig 2B)** (cardiac muscle: Control (*n* = 6) vs. DOB (*n* = 6): 2.2 ± 0.4% vs. 5.6 ± 0.7%, *P* < 0.01; masseter muscle: Control (*n* = 6) vs. DOB (*n* = 6): 2.6 ± 0.2% vs. 5.2 ± 0.9%, *P* < 0.05). However, CB did not alter the area of fibrosis in cardiac muscle or masseter muscle (cardiac muscle: Control (*n* = 6) vs. CB (*n* = 6): 2.2 ± 0.4% vs. 3.1 ± 0.2%, *P* = NS; masseter muscle: Control (*n* = 6) vs. CB (*n* = 6): 2.6 ± 0.2% vs. 3.0 ± 0.5%, *P* = NS).

These data indicate that fibrosis in cardiac and masseter muscles is mediated through the activation of β_1_-AR, but not β_2_-AR.

### Effects of CB and DOB in other muscles

We examined the effects of DOB on other skeletal muscles, TA (fast-twitch) and SOL (slow-twitch), by measuring the muscle mass per tibia length ratio. We also performed HE staining and compared the results with those derived from the masseter muscle (fast-twitch) **(S2A Fig and S2B Fig)**. The TA muscle mass per tibia length ratio was not different between the Control and DOB group, but it was significantly greater in the CB group than that in the Control, as in the case of masseter muscle. Also, the area of fibrosis was greater in the DOB group than in the CB group, as in the case of masseter muscle **(S2A Fig)**. Conversely, the SOL muscle mass per tibial length ratio was similar among the Control, DOB and CB groups, and no abnormal organization, such as fibrosis or muscle rupture, was observed **(S2B Fig)**. Importantly, these results are consistent with the idea that activation of β_1_-AR-mediated cAMP signaling with DOB might induce abnormality in fast-twitch muscle, but not in slow-twitch muscle, probably due to increased PDE4-mediated cAMP hydrolysis in SOL compared to masseter muscle or TA, resulting in a reduced cAMP concentration to activate the downstream β_1_-AR-mediated cAMP signaling [12].

### Myocyte apoptosis was similarly and significantly increased by DOB in both cardiac and masseter muscles

We also examined the effects of DOB and CB on myocyte apoptosis in cardiac **(Fig 2C)** and masseter muscles **(Fig 2D)** by means of TUNEL staining. Myocyte apoptosis in cardiac and masseter muscles was significantly increased by DOB (cardiac muscle: Control (*n* = 6) vs. DOB (*n* = 6): 0.03 ± 0.01 vs. 1.2 ± 0.5%, *P* < 0.01; masseter muscle: Control (*n* = 6) vs. DOB (*n* = 6): 0.01 ± 0.05 vs. 0.4 ± 0.08%, *P* < 0.05), whereas CB had no effect (cardiac muscle: Control (*n* = 6) vs. CB (*n* = 6): 0.03 ± 0.01 vs. 0.05 ± 0.02%, *P* = NS; masseter muscle: Control (*n* = 6) vs. DOB (*n* = 6): 0.08 ± 0.05 vs. 0.04 ± 0.04%, *P* = NS) **(Fig 2C and 2D)**.

For some samples, we performed TUNEL staining (green) with simultaneous detection of dystrophin with antibody (red) for muscle membrane identification and visualization of nuclei with Hoechst (blue) by using confocal microscopy, and confirmed that apoptotic cells identified as TUNEL-positive nuclei by light microscopy were located inside the myofibers **(S2C Fig)**.

These results, together with Masson-trichrome staining **(Fig 2A and 2B)**, indicate that fibrosis and apoptosis in both cardiac and masseter muscles are mediated through the activation of β_1_-AR, but not β_2_-AR.

### Apoptosis was significantly increased in C2C12 cells by DOB

We examined the effects of DOB and CB on apoptosis using differentiated murine myoblast C2C12 cells in vitro [44]. For the induction of myotube differentiation, cells were cultured in DMEM supplemented with 2% horse serum. We first examined the expression of β_1_- and β_2_-ARs by western blotting and confirmed that both expressions reached maximum at 5 days (Day 5) after the induction of cell differentiation **(S3A Fig)**.

We thus treated C2C12 cells with DOB or CB at 10^−4^ M on Day 4, incubated them for 24 hr, and then (Day 5) performed PI staining with immunohistochemistry for simultaneous detection of myosin with an anti-myosin heavy chain antibody (green) and Hoechst staining (blue) for visualization of nuclei **(S4A–S4C Fig)** [43]. PI-positive cells, which were also stained with an anti-myosin heavy chain antibody, were significantly increased by the DOB treatment (Control (*n* = 3) vs. DOB (*n* = 3): 2.2 ± 0.8 vs. 16.5 ± 1.2, *P* < 0.01), but not by the CB treatment (Control (*n* = 3) vs. CB (*n* = 3): 2.2 ± 0.8 vs. 4.4 ± 0.1, *P* = NS) **(S4D Fig)**.

We also examined the expression of cleaved caspase-8, an apoptosis-related cysteine protease [47], and found that it was significantly increased by the DOB treatment (Control (*n* = 4) vs. DOB (*n* = 6); 100 ± 7.4 vs. 153 ± 13%, *P* < 0.05), but not by the CB treatment (Control (*n* = 4) vs. CB (*n* = 6); 100 ± 7.4 vs. 119 ± 14%, *P* = NS) **(S3B Fig)**. These data were consistent with the in vivo studies shown in **Fig 2C** and **2D**.

### The number of microvessels per masseter myocyte was significantly decreased by DOB

Angiogenesis and hypertrophic response are closely associated in both skeletal muscle [48] and cardiac muscle [22,36]. We thus performed double immunostaining for dystrophin (red) and CD31 (green), and examined the number of microvessels per masseter myocyte in mice treated with DOB or CB **(S5A–S5C Fig)**. There was no difference in the number of microvessels between the Control and CB group (Control (*n* = 3) vs. CB (*n* = 3): 4.5 ± 0.2 vs. 4.6 ± 0.3, *P* = NS). However, the number was significantly decreased in the DOB group (Control (*n* = 3) vs. DOB (*n* = 3): 4.5 ± 0.2 vs. 1.6 ± 0.2, *P* < 0.01) **(S5D Fig)**.

These data suggest that activation of β_1_-AR signaling might alter the number of microvessels in masseter muscle, and might also be associated with the blunted hypertrophic response of masseter muscle to the DOB treatment.

### Ca^2+^ sensitivity of force and ATPase activity was not altered in DOB-treated mice

Cardiac as well as skeletal muscle function responds to a variety of changes in myocyte biology, including structural changes, altered Ca^2+^ handling, disrupted energetics, and modifications in myofibrillar function [49,50]. The Ca^2+^ sensitivity of force and ATP activity is regulated through the protein kinase A- or/and protein kinase C-mediated phosphorylation of myofilament protein in cardiac as well as skeletal muscle [51,52], but the effects of chronic β_1_- or β_2_-AR activation on calcium sensitivity in skeletal muscle have not been reported. We thus hypothesized that myofibrillar function might be altered in DOB-treated skinned masseter muscle.

As shown in **S6 Fig**, the Ca^2+^-activated isometric force (*lower trace*) and the decrease of ATPase activity, measured in terms of the consumption rate of nicotinamide adenine dinucleotide (NADH) (*upper trace*) were simultaneously recorded in a skinned masseter muscle strands.

The curves representing the force-pCa relationship **(S7A Fig)** and the ATPase activity-pCa relationship **(S7B Fig)** in the Control, DOB and CB groups at the Ca^2+^ concentration of 6.1, 5.8, 5.5, 5.1 and 4.6 (expressed as pCa = -log [Ca^2+^]) showed no leftward or rightward shift, suggesting that the Ca^2+^ sensitivity of force and ATPase activity were similar among the three groups. This might be due to the balance between the opposing effects of PKA (decrease of Ca^2+^ sensitivity; rightward shift) and Epac (increase of Ca^2+^ sensitivity; leftward shift) [53].

We also calculated the average pCa producing 50% force or ATPase activity, i.e., pCa_50_, and the Hill coefficients, by fitting the data of Ca^2+^-activated force and Ca^2+^-activated ATPase activity within the pCa range from 6.1 to 4.6 for the three groups **(S7C Fig and S7D Fig)**. Average pCa_50_ values (Ca^2+^ concentration required for half-maximal effect) of isometric force **(S7C Fig*left*)** and ATPase activity **(S7C Fig*right*)** were not significantly different among the three groups. Also, there were no significant differences of Hill coefficient (isometric force and ATPase activity) among the three groups **(S7D Fig)**

### Maximal isometric force was decreased in DOB-treated mice

**Fig 3:**
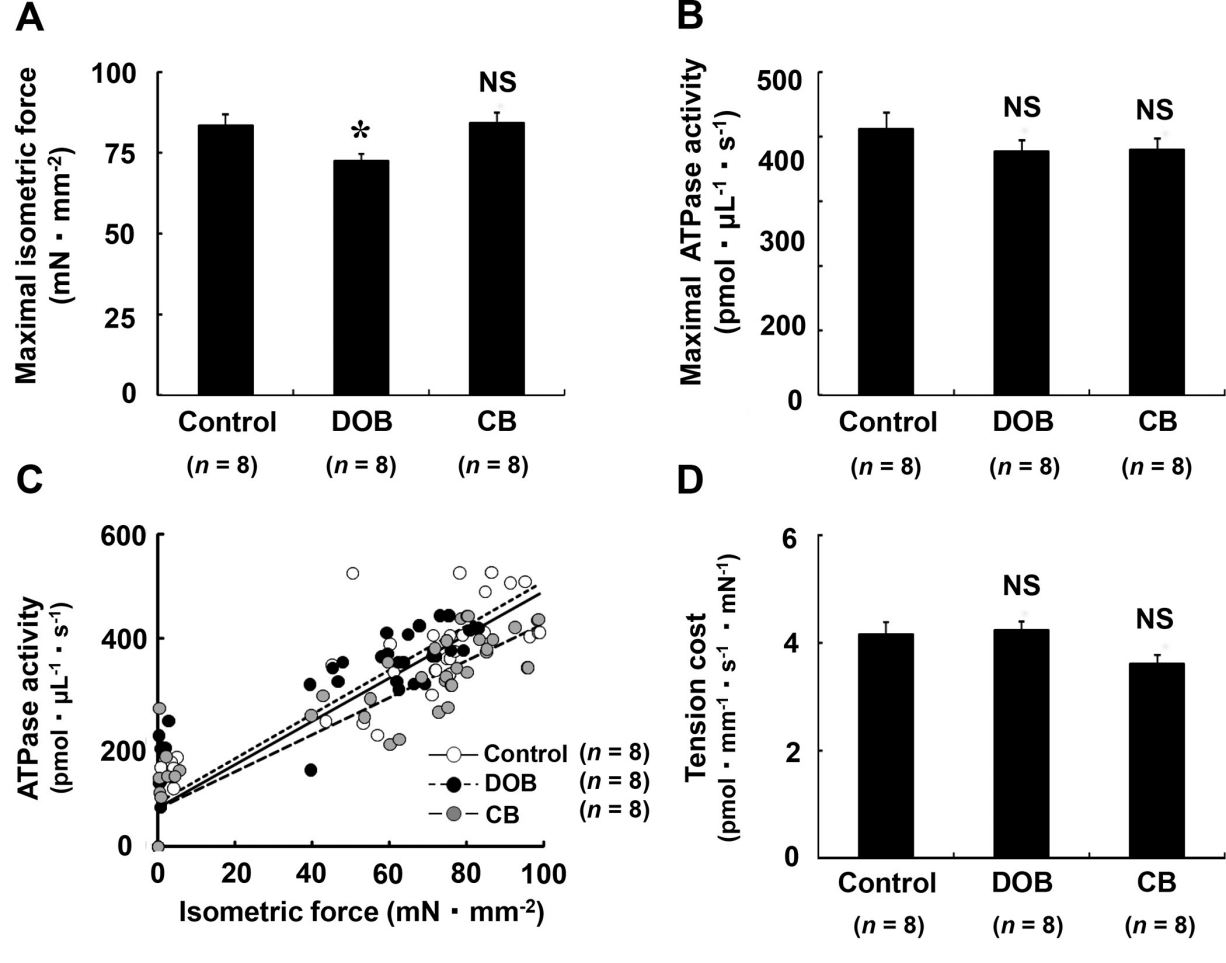
Effects of DOB and CB on maximal isometric force and ATPase activity and tension cost. **(A)** Maximal isometric force at pCa 4.6 (saturating [Ca^2+^]) was significantly smaller in the DOB group than in the Control (*n* = 8 each). **(B)** Maximal ATPase activity at pCa 4.6 in the DOB group or the CB group showed no significant differences from the Control (*n* = 8 each). **(C)** Relationship between ATPase activity and isometric force in the skinned masseter preparations from the Control, DOB, and CB groups. The average ATPase activity values at pCa 6.1, 5.8, 5.5, 5.1 and 4.6 were plotted against the corresponding average force values. The data points of the three groups (open circle, Control; closed circle, DOB; gray circle, CB) were fitted by linear regression as indicated (solid line, Control; dashed line DOB; long dashed line CB). The formulae of the regression lines are y = 4.16x + 74.28, r = 0.91 (Control), y = 4.24x + 84.38, r = 0.91 (DOB), y = 3.56x + 73.37, r = 0.9 (CB), (r: correlation coefficient). Slopes of the regression lines (ATPase activity/isometric force) indicate the tension cost. **(D)** The average tension cost in the DOB group or the CB group showed no significant difference from the Control (*n* = 8 each). CA; cardiac muscle, MA; masseter muscle.

We next compared the isometric force and ATPase activity at the maximal level of Ca^2+^ activation (pCa = 4.6) in the Control, DOB and CB groups **(Fig 3)**. Maximal isometric force was significantly smaller in the DOB group than in the Control (Control (*n* = 8) vs. DOB (*n* = 8): 84 ± 3.4 vs. 72 ± 2.3 mN/mm^2^, *P* < 0.05), but no significant decrease was observed in CB mice (Control (*n* = 8) vs. CB (*n* = 8): 84 ± 3.4 vs. 84 ± 3.3 mN/mm^2^, *P* = NS) **(Fig 3A)**. In marked contrast, maximal ATPase activity was similar among the three groups (*n* = 8 each) **(Fig 3B)**. In order to clarify the myofibrillar energy utilization in masseter muscle, we also examined the tension cost, i.e., ATPase activity/isometric force ratio, measured as the slope of the regression lines in the relationship between ATPase activity and isometric force **(Fig 3C)** and the values were similar among the three groups (*n* = 8 each) **(Fig 3D)**.

### Akt signaling was significantly increased by CB but not by DOB in both cardiac and masseter muscles

**Fig 4:**
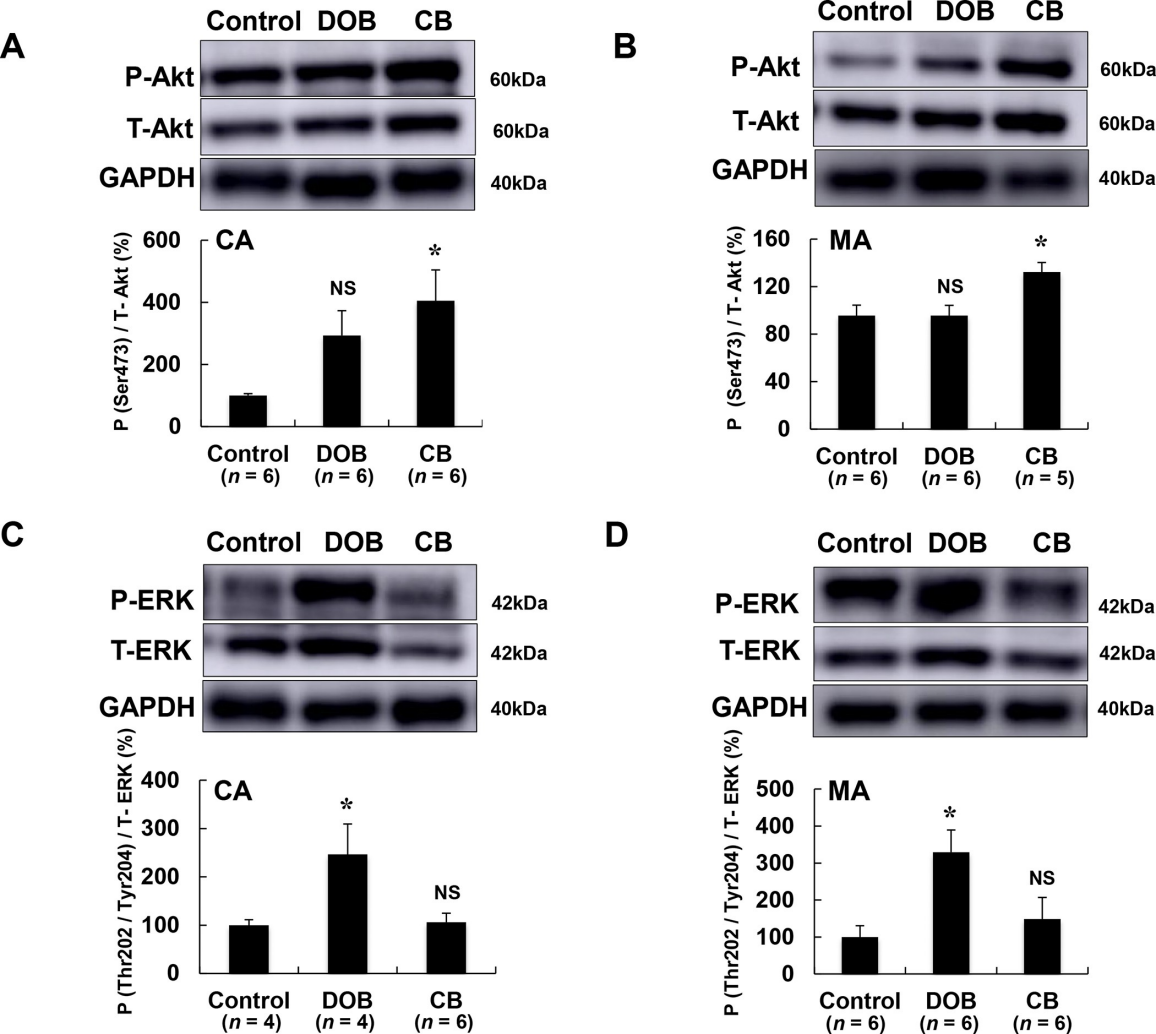
Effects of DOB and CB on Akt and ERK1/2 signaling pathways in cardiac and masseter muscles. **(A)** Akt phosphorylation (Ser-473) of cardiac muscle was significantly increased in the CB group (*P < 0.05 vs. Control). It was also increased, though without statistical significance, in the DOB group. **(B)** Akt phosphorylation (Ser-473) of masseter muscle was significantly increased in the CB group (*P < 0.05 vs. Control), but not in the DOB group. **(C)** ERK1/2 phosphorylation (Thr-202/Tyr-204) in cardiac muscle was significantly increased in the DOB group (*P < 0.05 vs. Control), but not in the CB group. **(D)** ERK1/2 phosphorylation (Thr-202/Tyr-204) in masseter muscle was also significantly increased in the DOB group (*P < 0.05 vs. Control), but not in the CB group. CA; cardiac muscle, MA; masseter muscle.

We then examined the phosphorylation of Akt in cardiac **(Fig 4A)** and masseter muscles **(Fig 4B)**, since this is known to be involved in cell-protective [9,10] and hypertrophic pathways [54,55] in the heart.

Akt phosphorylation (Ser-473) of cardiac muscle was significantly increased in the CB group (Control (*n* = 6) vs. CB (n = 6): 100 ± 6.0 vs. 405 ± 99%, *P* < 0.05). Although it was also increased in the DOB group, this was not statistically significant (*P* = NS vs. Control, *n* = 6 each) **(Fig 4A)**. Akt phosphorylation (Ser-473) of masseter muscle was also significantly increased in the CB group (Control (*n* = 6) vs. CB (*n* = 5): 100 ± 8.4 vs. 132 ± 7.9 mg *P* < 0.05), but not in the DOB group (Control (*n* = 6) vs. DOB (*n* = 6): 100 ± 8.4 vs. 96 ± 8.7 mg *P* = NS) **(Fig 4B)**.

These data suggest that induction of hypertrophy by CB in both cardiac and masseter muscle might be mediated, at least in part, through the activation (phosphorylation at Ser-473) of Akt.

### ERK1/2 activation was significantly increased by DOB but not by CB in both cardiac and masseter muscles

ERK1/2 activation is known to be involved in the development of cardiac fibrosis [56,57]. We thus examined the amount of phospho-ERK1/2 (Thr-202/Tyr-204) in cardiac **(Fig 4C)** and masseter muscles **(Fig 4D)** of DOB- or CB-treated mice. ERK1/2 phosphorylation (Thr-202/Tyr-204) in cardiac muscle was significantly increased in the DOB group (Control (*n* = 4) vs. DOB (*n* = 4): 100 ± 11 vs. 246 ± 63%, *P* < 0.05), but not in the CB group (Control (*n* = 4) vs. CB (*n* = 6): 100 ± 11 vs. 106 ± 19%, *P* = NS) **(Fig 4C)**. ERK1/2 phosphorylation (Thr-202/Tyr-204) in masseter muscle was also significantly increased in the DOB group (Control (*n* = 6) vs. DOB (*n* = 6): 100 ± 31 vs. 329 ± 60%, *P* < 0.05), but not in the CB group (Control (*n* = 6) vs. CB (*n* = 6): 100 ± 31 vs. 149 ± 58%, *P* = NS) **(Fig 4D)**.

These data suggest that DOB-mediated fibrosis in both cardiac and masseter muscle might be mediated, at least in part, through activation of ERK1/2 signaling.

### BAX expression was significantly increased by DOB in both cardiac and masseter muscles

**Fig 5:**
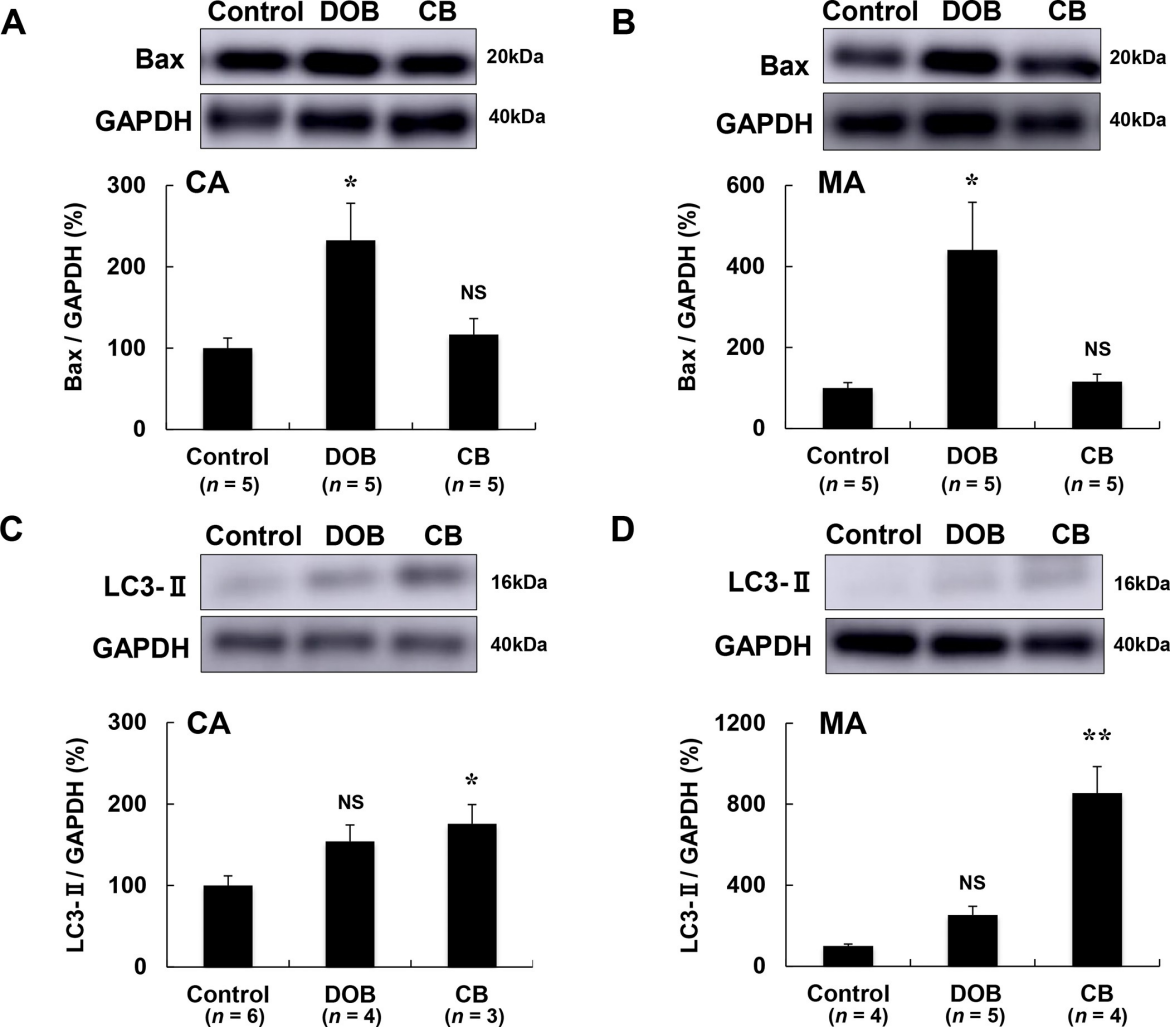
Effects of DOB and CB on expression of BAX and LC3 in cardiac and masseter muscles. **(A-B)** BAX expression was significantly increased in the DOB group, but not in the CB group, in both cardiac **(A)** and masseter **(B)** muscles (*P < 0.05 each vs. Control). **(C-D)** LC3-II expression was significantly increased in the CB group, but not in the DOB group, in both cardiac **(C)** and masseter muscles **(D)** (*P < 0.05 or **P < 0.01 each vs. Control). MA; masseter muscle.

We also examined changes of BAX, an accelerator of apoptosis, in cardiac **(Fig 5A)** and masseter muscle **(Fig 5B)** and found that its expression was significantly increased by DOB (cardiac muscle: Control (*n* = 5) vs. DOB (*n* = 5): 100 ± 12 vs. 232 ± 102%, *P* < 0.05; masseter muscle: Control (*n* = 5) vs. DOB (*n* = 5): 100 ± 13 vs. 441 ± 117%, *P* < 0.05), but not by CB (*P* = NS for both cardiac and masseter muscles vs. Control).

### Autophagy was induced by CB but not by DOB in cardiac and masseter muscles

We next investigated the effects of DOB and CB treatment on autophagy in the cardiac and masseter muscles, because autophagy is important to maintain muscle homeostasis physiologically and in response to stress [58]. Also, augmenting autophagy was reported to be protective [59], while suppressing autophagy was reported to be deleterious to the heart. We thus hypothesized that autophagy might be induced after β_2_-AR stimulation with CB, but not after β_1_-AR stimulation with DOB, in cardiac and skeletal muscle.

LC3-II, an autophagosome marker, was significantly increased in both cardiac **(Fig 5C)** and masseter muscles **(Fig 5D)** of the CB group (cardiac muscle: Control (*n* = 6) vs. CB (*n* = 3): 100 ± 12 vs. 176 ± 24%, *P* < 0.05; masseter muscle: Control (*n* = 4) vs. CB (*n* = 4): 100 ± 9 vs. 854 ± 263%, *P* < 0.01). In contrast, LC3-II expression was not significantly increased in either cardiac **(Fig 5C)** or masseter **(Fig 5D)** muscle of the DOB group.

These data suggest that activation of autophagy might be induced efficiently by activation of β_2_-AR rather than β_1_-AR in both cardiac and masseter muscles [60,61].

### mTORC1 was activated by CB and mTORC2 was activated by DOB in masseter muscle

mTOR phosphorylation at serine 2448 (mTORC1) is regulated by PI3-Akt signaling and mTOR phosphorylation at serine 2481 (mTORC2) is regulated by the cAMP-PKA pathway in skeletal muscle [11,17]. Also, PI3-Akt signaling is known to be protective and cAMP-PKA signaling is deleterious for cardiac muscle [32,55]. We thus hypothesized that mTOR phosphorylation at serine 2448 (mTORC1) might be mediated by β_2_-AR stimulation with CB and mTOR phosphorylation at serine 2481 (mTORC2) might be mediated by β_1_-AR stimulation with DOB in masseter muscle.

**Fig 6:**
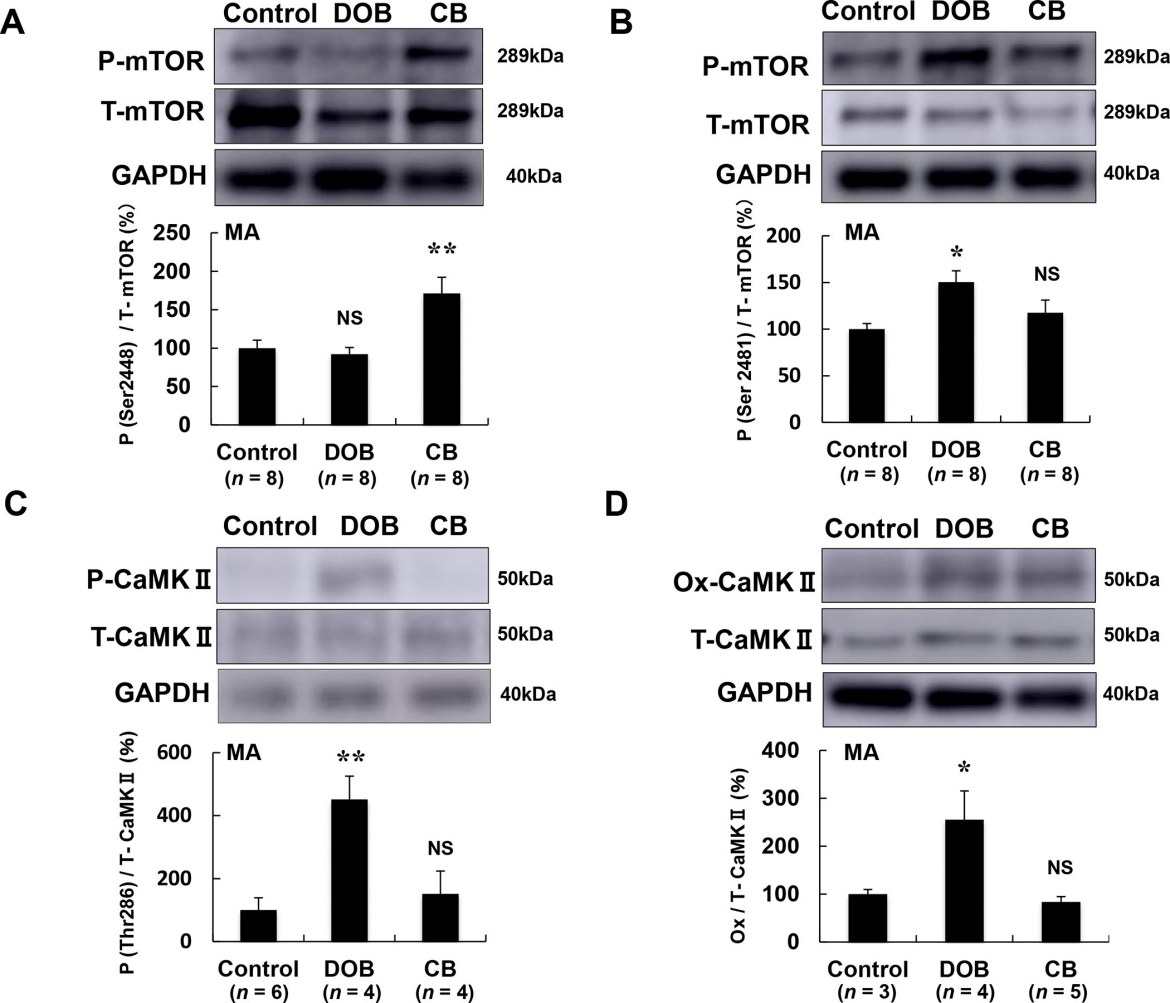
Effects of DOB and CB on mTORC1/C2 and CaMKII signaling pathways in masseter muscle. **(A)** In masseter muscle, mTOR phosphorylation on serine 2448, a specific marker of mTORC1 formation, was significantly increased in the CB group (**P < 0.01 vs. Control), but not in the DOB group in masseter muscle. **(B)** mTOR phosphorylation on serine 2481, a specific marker of mTORC2 formation, was significantly increased in the DOB group (*P < 0.05 vs. Control), but not in the CB group in masseter muscle. **(C-D)** CaMKII phosphorylation was significantly increased in the DOB group (**P < 0.01 vs. Control), but not in the CB group **(C)**. CaMKII oxidation was also significantly increased in the DOB group (*P < 0.05 vs. Control), but not in the CB group **(D)**. MA; masseter muscle.

We found that mTOR phosphorylation at serine 2448 was significantly increased in the CB group (Control (*n* = 8) vs. CB (*n* = 8): 100 ± 10 vs. 171 ± 21%, *P* < 0.01) but not in the DOB group (92 ± 8.9%, *P* = NS vs. Control, *n* = 8) **(Fig 6A)**. Conversely, mTOR phosphorylation at serine 2481 was significantly increased in the DOB group (Control (*n* = 8) vs. DOB (*n* = 8): 100 ± 6 vs. 150 ± 12%, *P* < 0.05) but not in the CB group (118 ± 13%, *P* = NS vs. Control, *n* = 8) **(Fig 6B)**.

These data suggest that masseter muscle fibrosis and apoptosis in DOB-treated mice might be associated with the activation of mTORC2 signaling.

### CaMKII phosphorylation and oxidation were significantly increased by DOB in masseter muscle

CaMKII is crucial for cardiac excitation-contraction coupling and its expression is increased in heart failure in animals and humans [62,63]. However, the role of CaMKII in skeletal muscle has not yet been examined. Thus, we examined the effects of DOB or CB on CaMKII phosphorylation at threonine 286 and CaMKII oxidation in masseter muscle.

CaMKII phosphorylation (Thr-286) was significantly increased in the DOB group (Control (*n* = 6) vs. DOB (*n* = 4): 100 ± 39 vs. 450 ± 74%, *P* < 0.01), but not in the CB group (151 ± 72%, *P* = NS vs. Control) **(Fig 6C)**. CaMKII oxidation was also significantly increased in the DOB group (Control (*n* = 3) vs. DOB (*n* = 4): 100 ± 9.6 vs. 255 ± 60%, *P* < 0.05), but not in the CB group (83 ± 11%, *P* = NS vs. Control) **(Fig 6D)**.

These data suggest that masseter muscle fibrosis and apoptosis might be mediated, at least in part, through the activation of CaMKII signaling.

### Ca^2+^ homeostasis was altered in DOB-treated mice

The maintenance of calcium (Ca^2+^) homeostasis during muscle contraction is requisite for optimal contractile function, and altered Ca^2+^ homeostasis might induce hyperactivation of calcineurin-NFAT signaling and calpain signaling [64,65].

**Fig 7:**
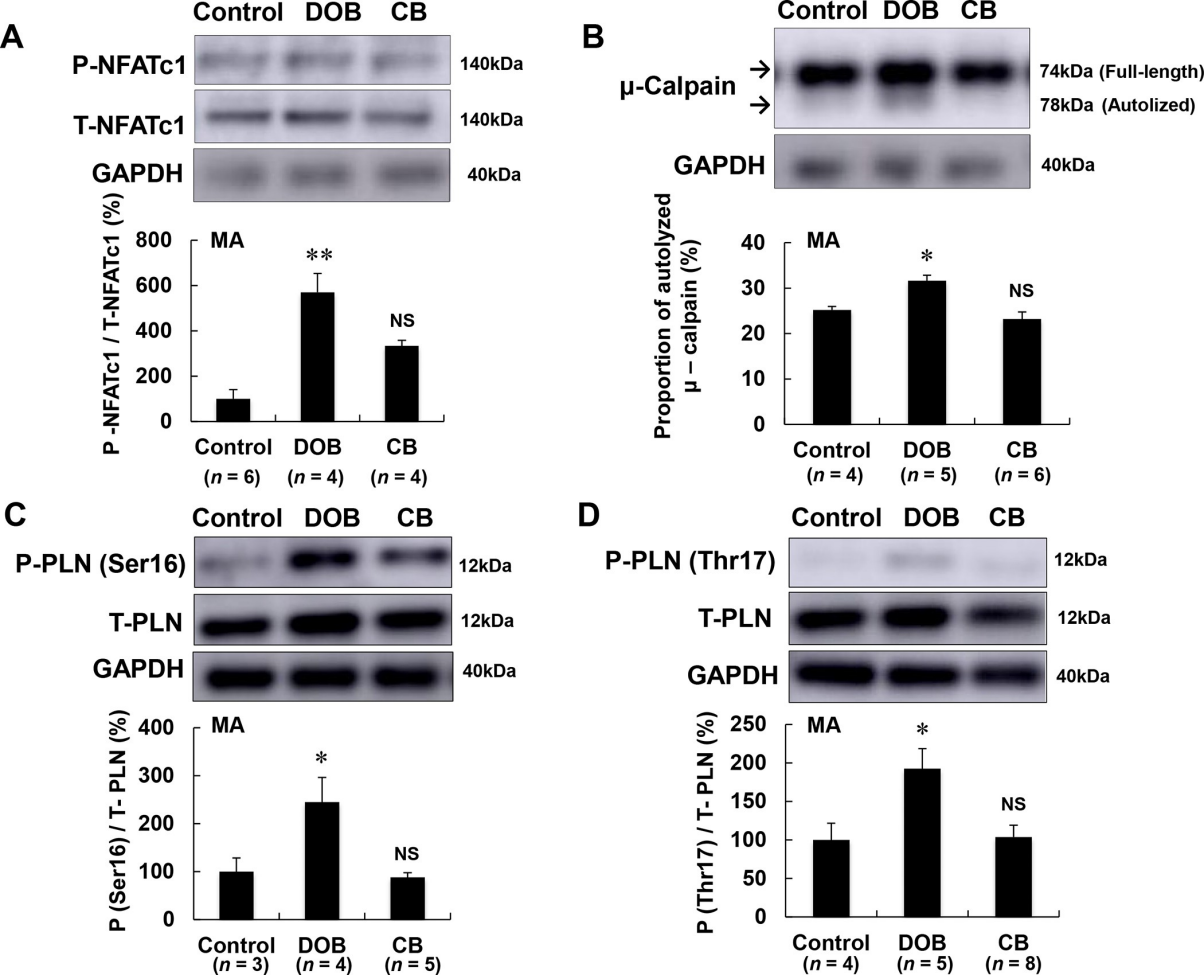
Effects of DOB and CB on Ca^2+^ homeostasis and PLN phosphorylation in masseter muscle. **(A)** NFATc1 phosphorylation on serine 259 was significantly greater in masseter muscle of the DOB group compared with the control group (*P < 0.05 vs. Control), but this was not the case in the CB group. **(B)** The calculated μ-calpain fractional activation index (i.e., percentage of autolysed calpain) was significantly increased in the DOB group (**P < 0.01 vs. Control), but not in the CB group. **(C)** PLN phosphorylation (Ser-16) was significantly increased in the DOB group (*P < 0.05 vs. Control), but not in the CB group. **(D)** PLN phosphorylation (Thr-17) was significantly increased in the DOB group (*P < 0.05 vs. Control), but not in the CB group.

We assessed the levels of Ca^2+^-handling proteins involved in Ca^2+^ homeostasis in skeletal muscle. We first examined the activation level of calcineurin-NFAT signaling in terms of phosphorylation level on serine 259 of NFATc1 [66], and found that it was significantly greater in masseter muscle of DOB mice than in control mice (Control (*n* = 3) vs. DOB (*n* = 3): 100 ± 40 vs. 570 ± 84%, *P* < 0.01), whereas this was not the case in CB-treated mice (Control (*n* = 3) vs. CB (*n* = 3): 100 ± 40 vs. 333 ± 24%, *P* = NS) **(Fig 7A)**.

Calpain is an intracellular Ca^2+^-activated cysteine protease, but the physiological roles of calpains are not well understood, although μ-calpain has been associated with skeletal muscle remodeling [14]. Calpains are present predominantly in their full-length, unautolysed/unactivated forms at rest. However, calpain appears in autolysed forms upon activation and measurement of these forms can be used to determine when in vivo induction of Ca^2+^-mishandling occurs in a Ca^2+^-dependent manner [14]. We thus examined μ-calpain activity by measuring the autolysis level in masseter muscle and found that μ-calpain autolysis was significantly greater in masseter muscle of DOB mice than in the control group (Control (*n* = 4) vs. DOB (*n* = 5): 25 ± 0.8 vs. 32 ± 1.2%, *P* < 0.05), whereas no significant increase was observed in CB mice (Control (*n* = 4) vs. CB (*n* = 6): 25 ± 0.8 vs. 23 ± 1.5%, *P* = NS) **(Fig 7B)**.

These data, together with the increased NFATc1 phosphorylation level in response to DOB treatment, indicated that calcium homeostasis was altered in masseter muscle of DOB-treated mice.

### PLN phosphorylation was significantly increased by DOB in masseter muscle

The importance of PLN regulation of SERCA function in cardiac muscle health and disease is well established [22]. Recently, several studies have indicated that it might also play an important role in the development of skeletal muscle disease [24,25]. We thus examined the effects of DOB and CB on PLN phosphorylation (Ser-16) **(Fig 7C)** and PLN phosphorylation (Thr-17) in masseter muscle **(Fig 7D)**.

PLN phosphorylation (Ser-16) was significantly increased in the DOB group (Control (*n* = 3) vs. DOB (*n* = 4): 100 ± 28 vs. 245 ± 52%, *P* < 0.05), but not in the CB group (Control (*n* = 3) vs. CB (*n* = 5): 100 ± 28 vs. 88 ± 9%, *P* = NS) **(Fig 7A)**. We further examined the PLN phosphorylation at Thr-17 and found that it was also significantly increased in the DOB group (Control (*n* = 4) vs. DOB (*n* = 5): 100 ± 22 vs. 192 ± 26%, *P* < 0.05), but not in the CB group (Control (*n* = 4) vs. CB (*n* = 8): 100 ± 22 vs. 104 ± 15%, *P* = NS) **(Fig 7B)**.

These data suggest that DOB-mediated masseter muscle fibrosis **(Fig 2B)** and apoptosis **(Fig 2D)** might be induced, at least in part, through increased PLN phosphorylation at serine 16 and threonine 17, as we previously showed to be the case in cardiac muscle [22].

## Discussion

In this study, we examined the effects of chronic β_1_-AR or β_2_-AR stimulation on cardiac and masseter muscles of mice, using a selective β_1_-AR agonist (DOB) and a selective β_2_-AR agonist (CB). We have demonstrated that activation of β_1_-AR signaling plays an important role in the development of myocyte apoptosis and fibrosis not only in cardiac muscle, but also in skeletal muscle.

In this study, cardiac hypertrophy in terms of cardiac muscle mass/tibial length ratio was significantly increased by approximately 21% in the DOB group (2 mg/kg/day for 1 week) and by approximately 16% in the CB group (2 mg/kg/day for 1 week) **(Fig 1B)**. In previous studies in mice, the non-specific β-AR agonist isoproterenol (30 mg/kg/day for 1 week) induced cardiac hypertrophy by approximately 17% [67], while the β_2_-AR specific agonist CB (2 mg/kg/day for 4 weeks) also induced cardiac hypertrophy by approximately 17% [68], suggesting that hypertrophic response was similar to our study, even though the dose and treatment period were greater.

The cardiac hypertrophy found in this study, as well as in previous reports, suggest that the local tissue concentrations of DOB and CB used in this study would have been sufficient to activate β_1_-AR and β_2_-AR, respectively, in skeletal muscle, as well as in cardiac muscle. Thus, we think the experimental conditions used in this study might be appropriate to examine the role of β_1_-AR and β_2_-AR signaling of skeletal muscle in mice. So, we think the current findings in skeletal muscle exposed to β_1_-AR stimulation with DOB (2 mg/kg/day for 1 week) or β_2_-AR stimulation with CB (2 mg/kg/day for 1 week) properly reflect the roles of β_1_-AR and β_2_-AR signaling in skeletal muscle.

This study, together with previous studies, suggested that an endogenous β_1_-selective agonist norepinephrine, which is markedly increased in heart failure patients, induces deterioration of contractility of not only cardiac muscle [69,70], but also fast-twitch skeletal muscles such as masseter and TA muscles. On the other hand, β_2_-AR signaling is protective via the increase of muscle hypertrophy and contractility and the decrease of myocardial apoptosis not only in cardiac muscle, but also in skeletal muscle [71], indicating that β_1_-AR-mediated cAMP signaling in cardiac and fast-twitch skeletal muscle might play important roles in the induction of muscle dysfunction independently of their expression levels in each muscle, as demonstrated previously in TA and SOL [12].

**Fig 8:**
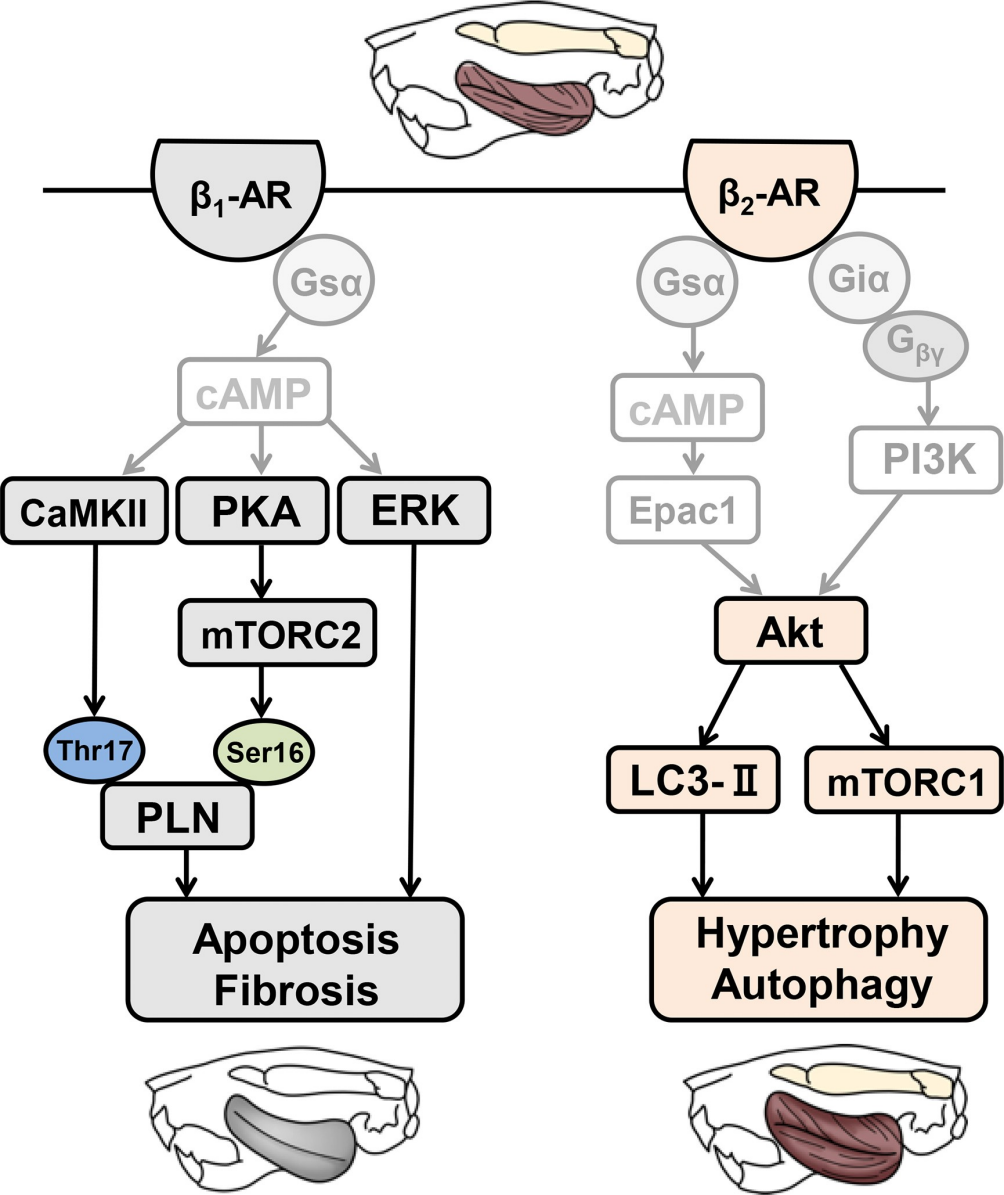
Schematic illustration of the proposed roles of β-AR signaling in masseter muscle. This scheme illustrates the proposed relationship of β_1_-AR and β_2_-AR signaling in masseter muscle. Chronic β_1_-AR stimulation with DOB induces muscle fibrosis and apoptosis with the increase of ERK1/2, CaMKII and mTORC2 activation (*left*). Chronic β_1_-AR stimulation with CB induces muscle hypertrophy and autophagy with increase of mTORC1 activation and LC3-II expression (*right*). Solid black lines represent findings in this study and solid grey lines represents findings reported previously (Lynch et al., 2008 [93], Ohnuki et al. 2014 [11], Ohnuki et al 2016 [12], Okumura et al. 2014 [22]).

Both β_1_-AR and β_2_-AR stimulate the classic Gsα-adenylyl cyclase-cAMP signaling cascade, but β_2_-AR couples to both Gsα and Giα proteins, activating bifurcated signaling pathways **(Fig 8)**. In the cardiac muscle, chronic β_1_-AR stimulation with DOB induced cardiac remodeling (hypertrophy, fibrosis and myocyte apoptosis) together with increased BAX expression and ERK1/2 phosphorylation (Thr-202/Tyr-204); this is known to be a reactive oxygen species (ROS)-mediated pathway resulting in cardiac injury [72,73]. On the other hand, chronic β_2_-AR stimulation with CB induced cardiac hypertrophy together with activation of Akt (Ser-473), which is a powerful promoter of cell survival and physiological hypertrophy [55,74].

In contrast to the cardiac muscle, chronic β_1_-AR stimulation with DOB did not induce hypertrophy of masseter muscle, but caused muscle fibrosis and myocyte apoptosis with increased BAX expression, ERK1/2 phosphorylation (Thr-202/Tyr-204) and CaMKII phosphorylation (Thr-286) **(Fig 8)**, though Akt phosphorylation (Ser-473) was not induced. On the other hand, chronic β_2_-AR stimulation with CB induced masseter muscle hypertrophy with activation of Akt (Ser-473), but did not induce myocyte apoptosis or fibrosis. We also identified a significant increase of LC3-II protein expression, an autophagosome marker, in masseter muscle of CB-treated mice, but not DOB-treated mice. Proper regulation of the autophagy flux is critical for skeletal muscle homeostasis in response to various stresses and physiological stimuli [58]. More recently, it has been suggested that β_2_-AR signaling might contribute to exercise training-mediated adaptations in insulin signaling and autophagy regulation through peroxisome-proliferator-activated receptor-γ coactivator-1α in skeletal muscle [75]. Taken together, these results indicate that β_2_-AR may be beneficial by promoting muscle hypertrophy and autophagy might be beneficial in response to various stresses **(Fig 8)**.

In order to examine the reason for the different effects of β_1_-AR stimulation in masseter muscle and cardiac muscle, we focused on mTOR, which is a downstream target of Akt and ERK1/2, and integrates a variety of environmental cues to regulate organismal growth and homeostasis [76]. mTOR assembles into two distinct multiprotein complexes, termed mTOR complex 1 (mTORC1) and mTORC2: it is phosphorylated at serine 2448 within the mTORC1 complex (associated with Raptor; regulatory associated protein of mTOR) and at serine 2481 within the mTORC2 complex (associated with Rictor; rapamycin insensitive companion of mTOR) [77]. It was recently shown that mTOR phosphorylation at serine 2448 (mTORC1) is regulated by PI3K-Akt signaling, while mTOR phosphorylation at serine 2481 (mTORC2) is regulated by the cAMP-PKA pathway in skeletal muscle [17].

In this study, mTOR phosphorylation (Ser-2448) was significantly increased by CB but not by DOB, while mTOR phosphorylation (Ser-2481) was significantly increased by DOB but not by CB. The experimental data in this study is consistent with the previous finding that mTORC1 signaling might regulate cell growth and proliferation in response to insulin, nutrients, and growth factors [17,76]. In contrast, much less is known about the role of mTORC2. Insofar as sympathetic tone increases with age, our data might indicate that activation of mTORC2 plays a pivotal role in the development of skeletal muscle weakness through the accumulation of skeletal muscle apoptosis and fibrosis induced via β_1_-AR stimulation during aging.

PLN is a well-known inhibitor of SERCA2a that maintain low levels of cytosolic Ca^2+^ in muscle and play a crucial role in muscle contraction [21]. The importance of PLN regulation of SERCA2a function in cardiac muscle health and disease is well established [78,79]. Indeed, decreased PLN phosphorylation, leading to a decrease of Ca^2+^ uptake by SERCA2a, is a central feature of heart failure [80,81], and decreased inhibition of SERCA2a by PLN ablation can prevent progression of heart failure [82,83]. Conversely, other groups found that decreased inhibition of SERCA2a through increased PLN phosphorylation might exaggerate heart failure and arrhythmogenic activity [84,85]. More recently, we have demonstrated that disruption of Epac1 protects the heart from chronic catecholamine stress, chronic pressure overload, and arrhythmogenic susceptibility through the inhibition of PLN phosphorylation (Ser-16 and Thr-17), together with decreased RyR2 phosphorylation (Ser-2808 and Ser-2814) [22]. However, the role of PLN phosphorylation in the development of skeletal muscle diseases remains poorly understood.

In this study, we found that chronic β_1_-AR stimulation did not induce muscle hypertrophy in masseter muscle, but it caused muscle fibrosis and apoptosis with an increase of PLN phosphorylation (Ser-16 and Thr-17), indicating that increase of β_1_-AR-mediated PLN phosphorylation might alter the Ca^2+^ leakage from sarcoplastic reticulum in skeletal muscle, as in the case in cardiac muscle [22].

Mouse and rabbit models overexpressing PLN in slow-twitch muscle fibers (PLN-TG) have been generated by attaching a PLN transgene to the β-myosin heavy chain promoter, which preferentially directs high levels of expression of slow-twitch muscle specific transgene. Importantly, PLN-TG mice show muscular dystrophy, including severe muscle wasting, fibrosis, fatty infiltration and muscle weakness [24,25].

β_2_-AR stimulation has been reported to evoke vasodilatation in skeletal muscle, including masseter muscle, and impaired β_2_-AR function is implicated in the maintenance of systemic arterial hypertension [86,87]. β_2_-AR-mediated vasodilatation is caused through the upregulation of endothelial nitric oxide synthase via the PI3-kinase-Akt pathway in mouse pulmonary artery or rat cerebral artery [88,89]. More importantly, cardiorespiratory function and cerebral blood oxygen influence cognitive function, physical performance, and rhythmic movement of the masseter muscle [90–92]. Also β_2_-AR-gene deletion in mice leads to impaired insulin secretion by pancreatic β-cells, indicating that impaired β_2_-AR function might alter insulin sensitivity and insulin signaling in skeletal muscle [27]. Thus, the β_2_-AR-mediated protective effects in masseter muscle described in our current study, as well as in previous reports, might be mediated via not only through direct mechanisms involving β_2_-AR in masseter muscle, but also in part via circulatory and/or hormonal indirect mechanisms.

As shown in **S2A Fig** and **S2B Fig**, we examined the effects of β_1_-AR stimulation with DOB on the tissue morphology of TA (fast-twitch) and SOL (slow-twitch) in terms of the muscle mass per tibial length ratio and HE staining. We found that β_1_-AR stimulation did not induce hypertrophy but induced fibrosis, as in the case of masseter muscle (fast-twitch muscle) [11,12]. These data suggest that activation of β_1_- and β_2_-AR signaling in **Fig 8** might be specific to fast-twitch muscles such as masseter and TA muscles.

Taken together with the previous findings, our results indicate for the first time that the β_1_-AR signaling pathway is a potential therapeutic target for the treatment of skeletal muscle wasting and weakness.

## Supporting information

S1 Fig S1 Fig PLOS ONE-3rd revised.pdf. and S1 Fig. (PDF)

S2 Fig S2 Fig PLOS ONE-3rd revised.pdf and S2 Fig. (PDF)

S3 Fig S3 Fig PLOS ONE-3rd revised.pdf and S3 Fig. (PDF)

S4 Fig S4 Fig PLOS ONE-3rd revised.pdf and S4 Fig. (PDF)

S5 Fig S5 Fig PLOS ONE-3rd revised.pdf and S5 Fig. (PDF)

S6 Fig S6 Fig PLOS ONE-3rd revised.pdf and S6 Fig. (PDF)

S7 Fig S7 Fig PLOS ONE-3rd revised.pdf and S7 Fig. (PDF)
