# Correction: Role of β-adrenergic signaling in masseter muscle

**DOI:** 10.1371/journal.pone.0216752

**Published:** 2019-07-16

**Authors:** The PLOS ONE Staff

The following information is missing from the Funding statement: MEXT-Supported Program for the Strategic Research Foundation at Private Universities S1511018 to Dr. Satoshi Okumura. Mitsui Life Social Welfare Foundation to Satoshi Okumura. An Academic Contribution from Pfizer Japan to Satoshi Okumura. The publisher apologizes for the error.

The Supporting Information figure legends for S1-S7 Fig are omitted from the manuscript. The authors have provided the S1-S7 Fig legends as an additional Supporting Information file. It can be viewed below.

## Supporting information

S1 Supporting information figure legends (PDF)
