# Supplementary figures and images for: Role of β-adrenergic signaling in masseter muscle
Source: PLoS One. 2019 Apr 15;14(4):e0215539. doi: 10.1371/journal.pone.0215539 (PMC6464212; doi:10.1371/journal.pone.0215539)

Fig S1

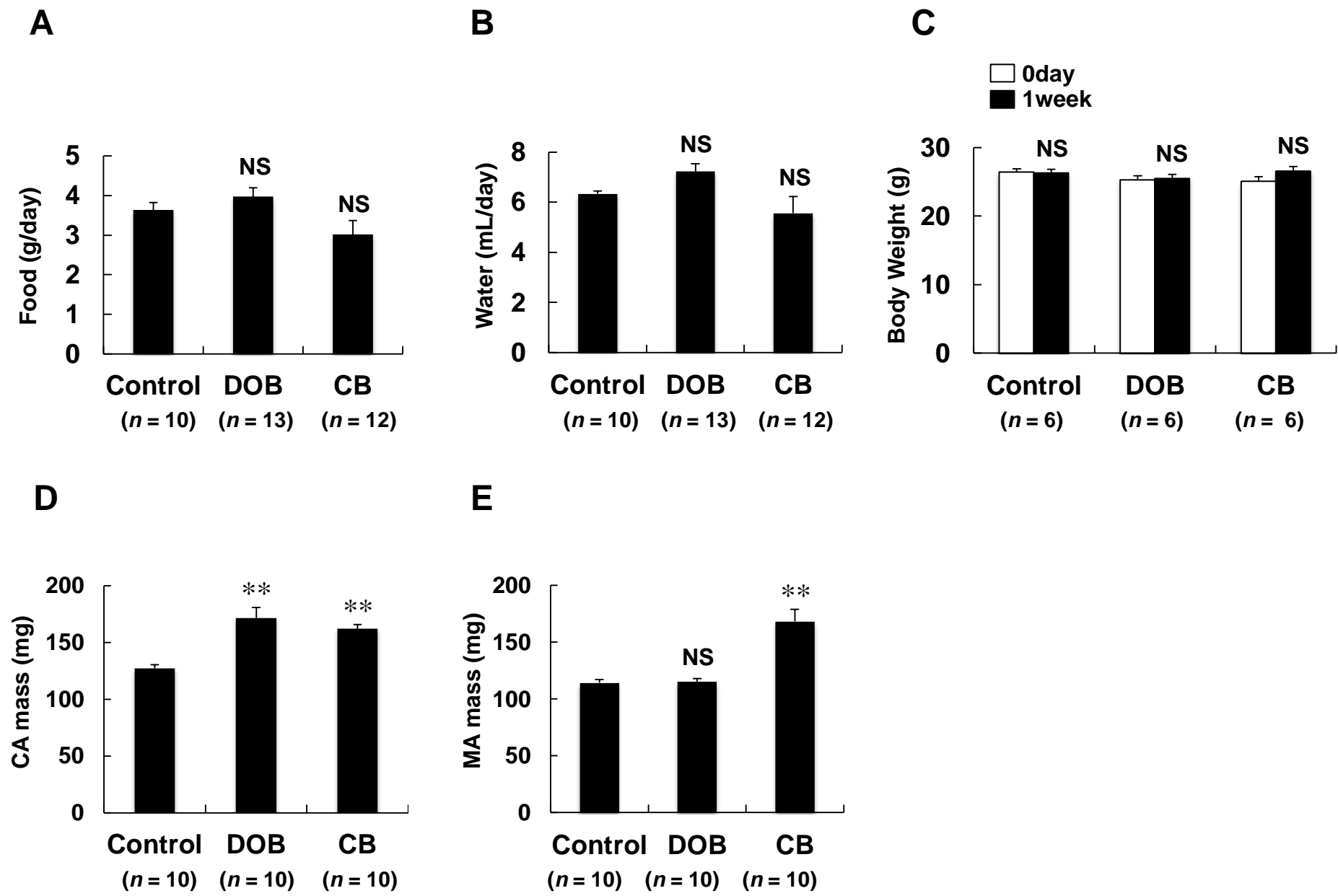

Supplement: S1 Fig — (PDF) [file pone.0215539.s001.pdf]

Fig S2

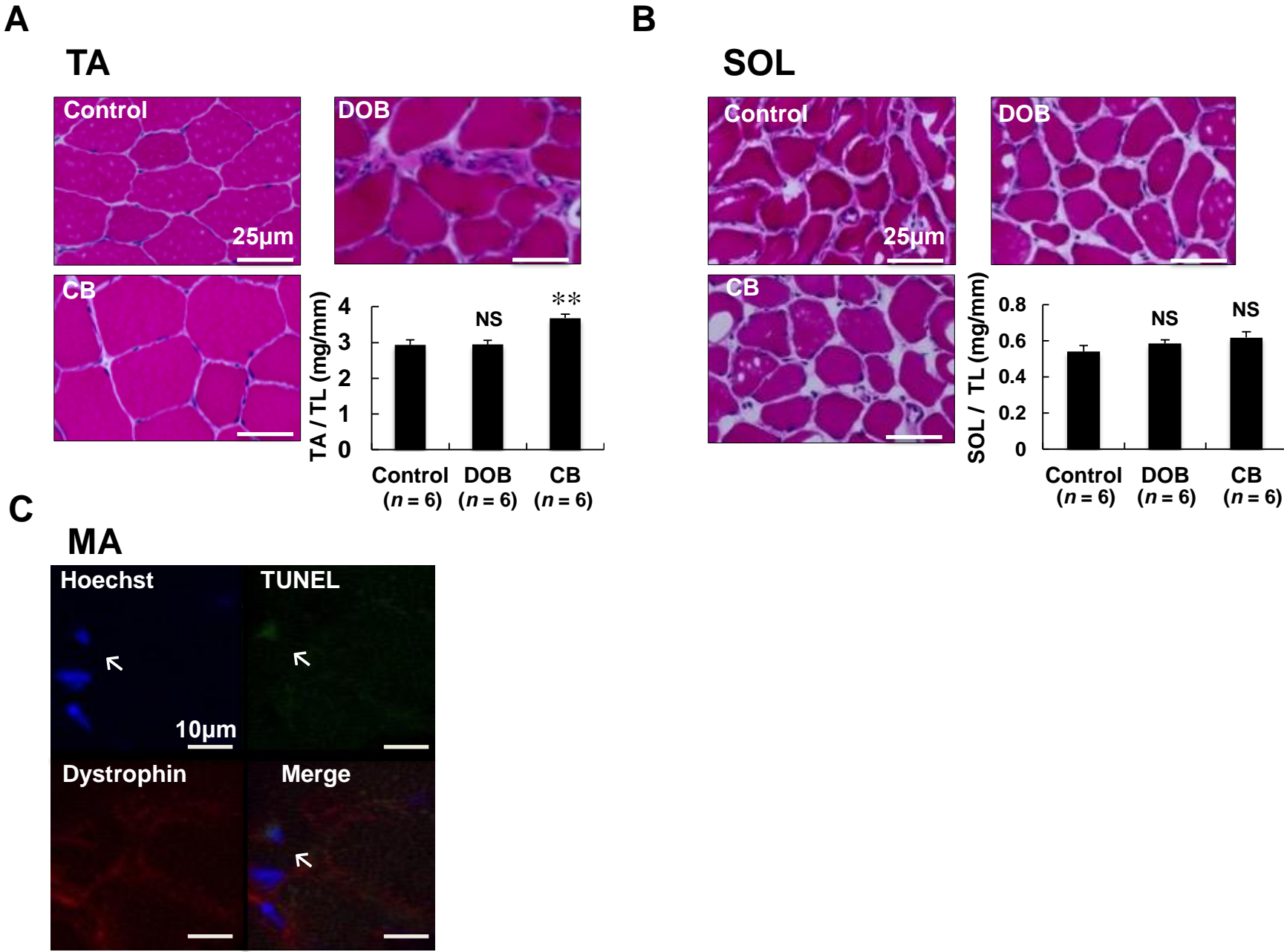

Supplement: S2 Fig — (PDF) [file pone.0215539.s002.pdf]

Fig S3

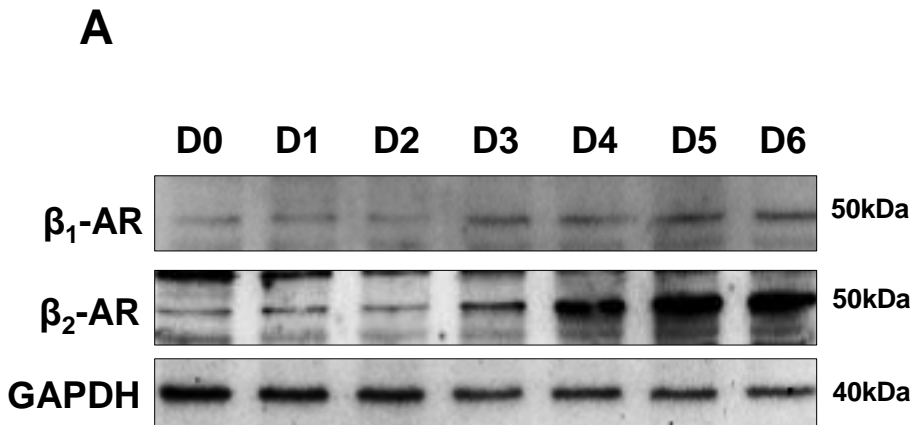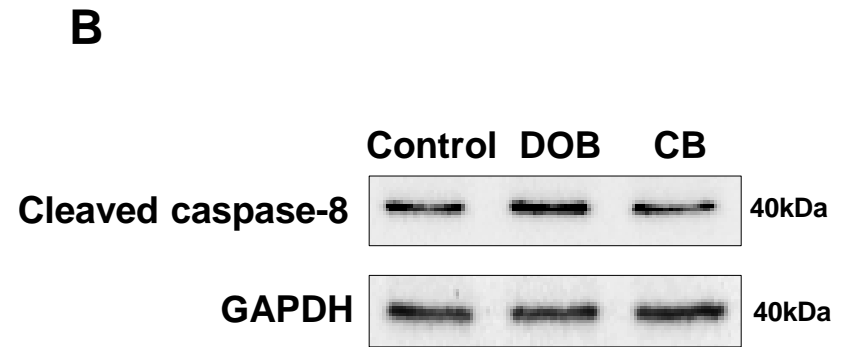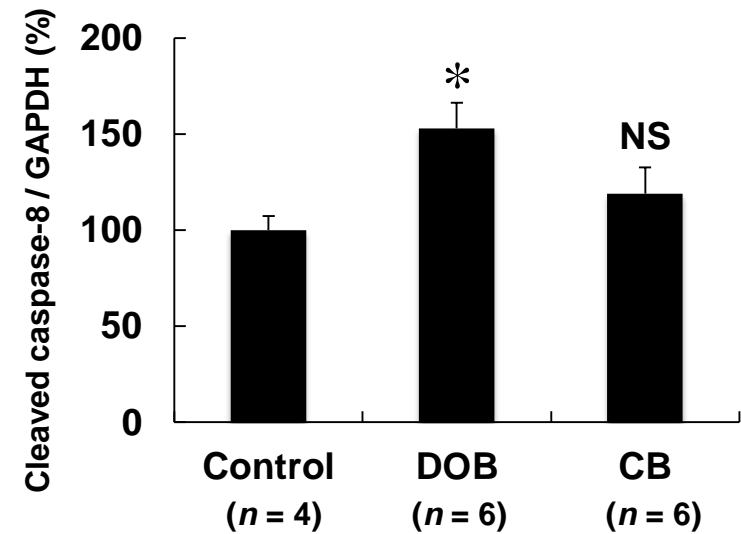

Supplement: S3 Fig — (PDF) [file pone.0215539.s003.pdf]

**Fig S4**

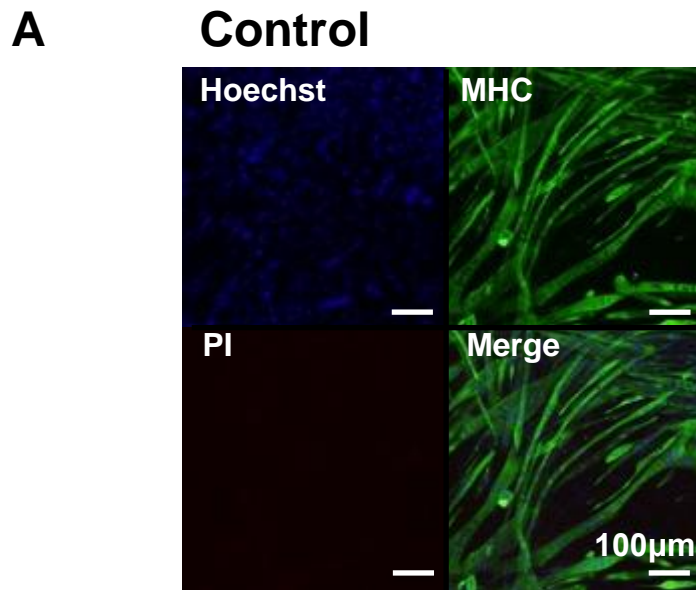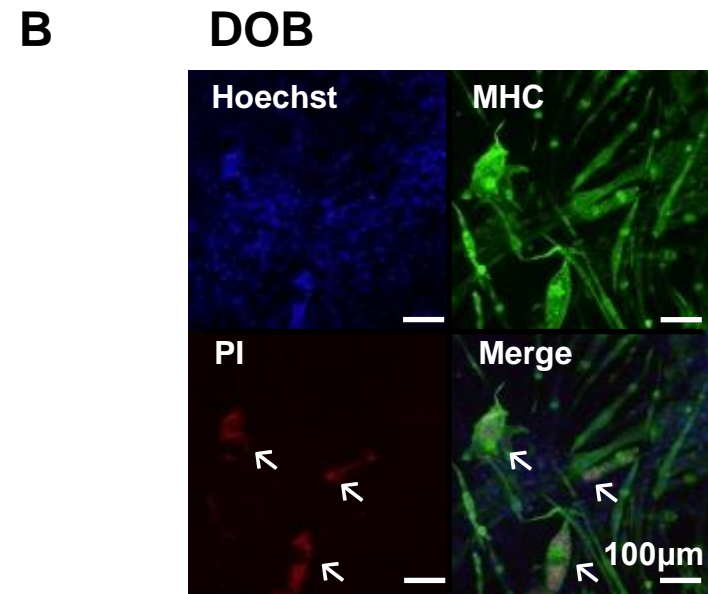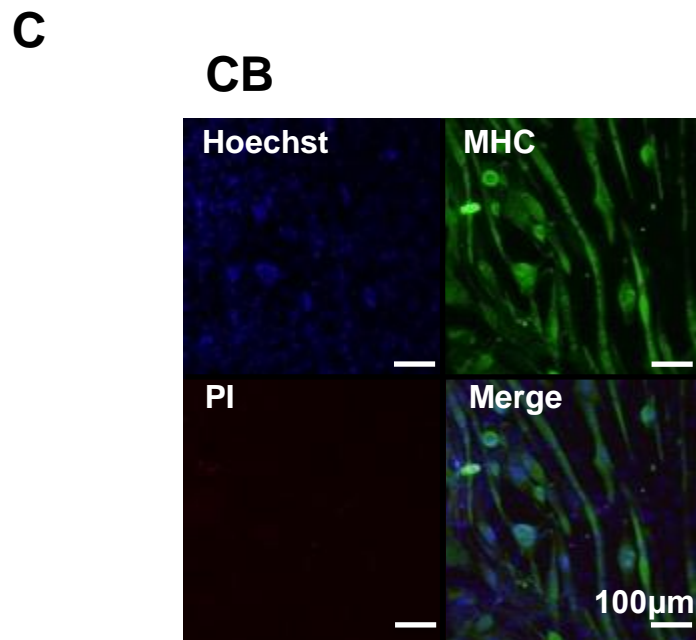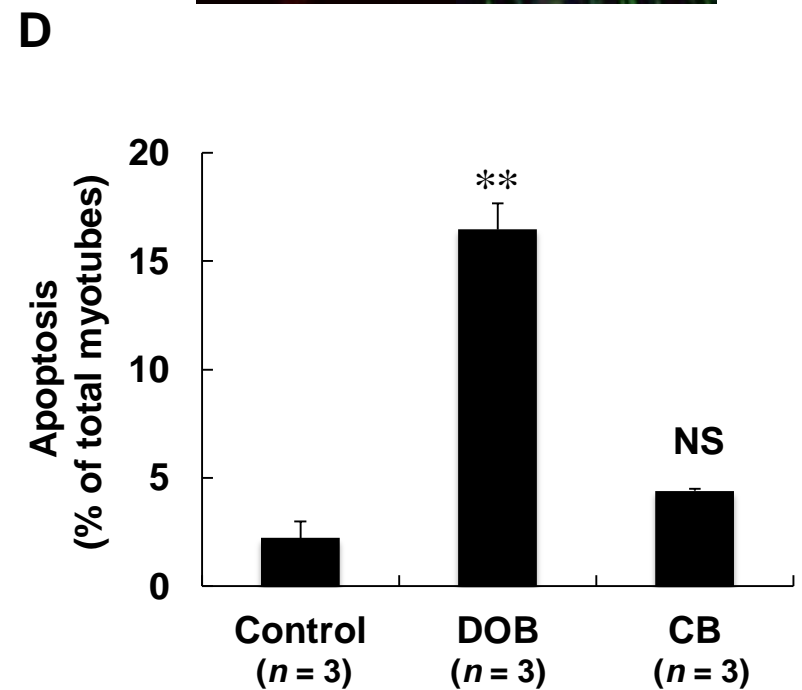

Supplement: S4 Fig — (PDF) [file pone.0215539.s004.pdf]

**Fig S5**

**A**

**Control**

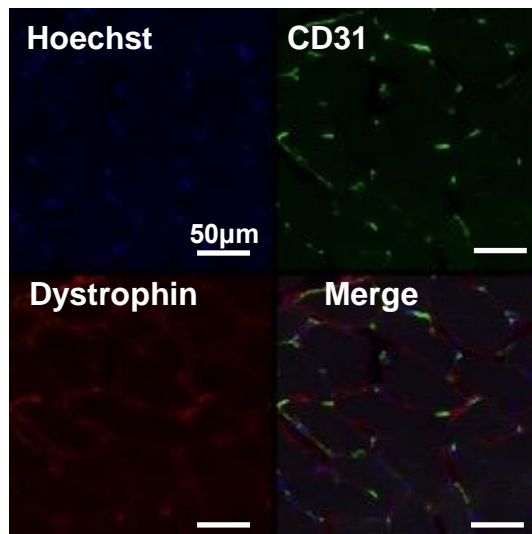

**B**

**DOB**

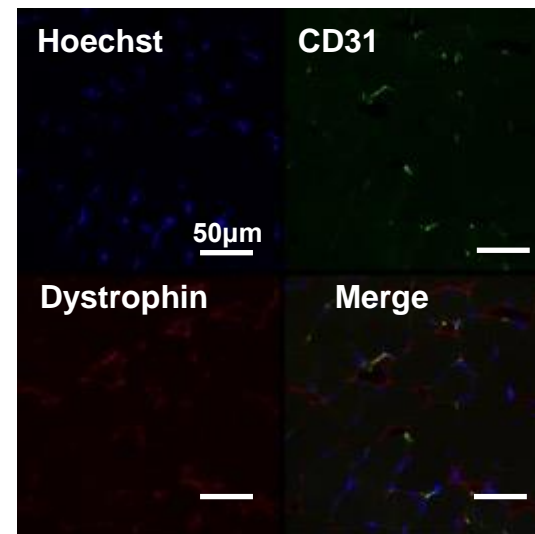

**C**

**CB**

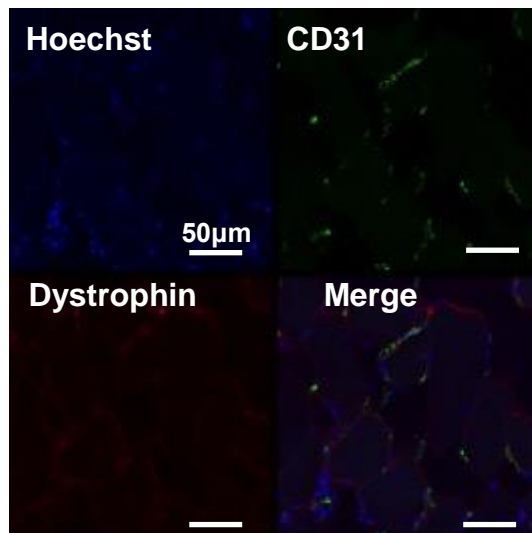

**D**

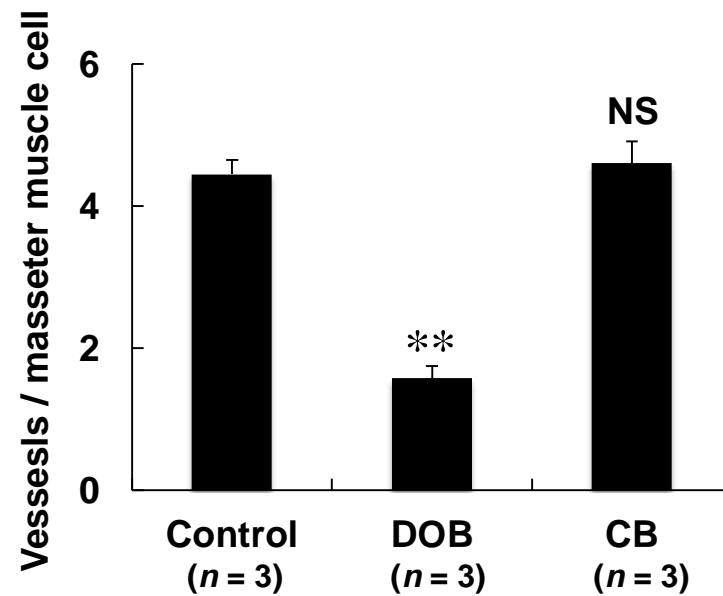

Supplement: S5 Fig — (PDF) [file pone.0215539.s005.pdf]

**Fig S6**

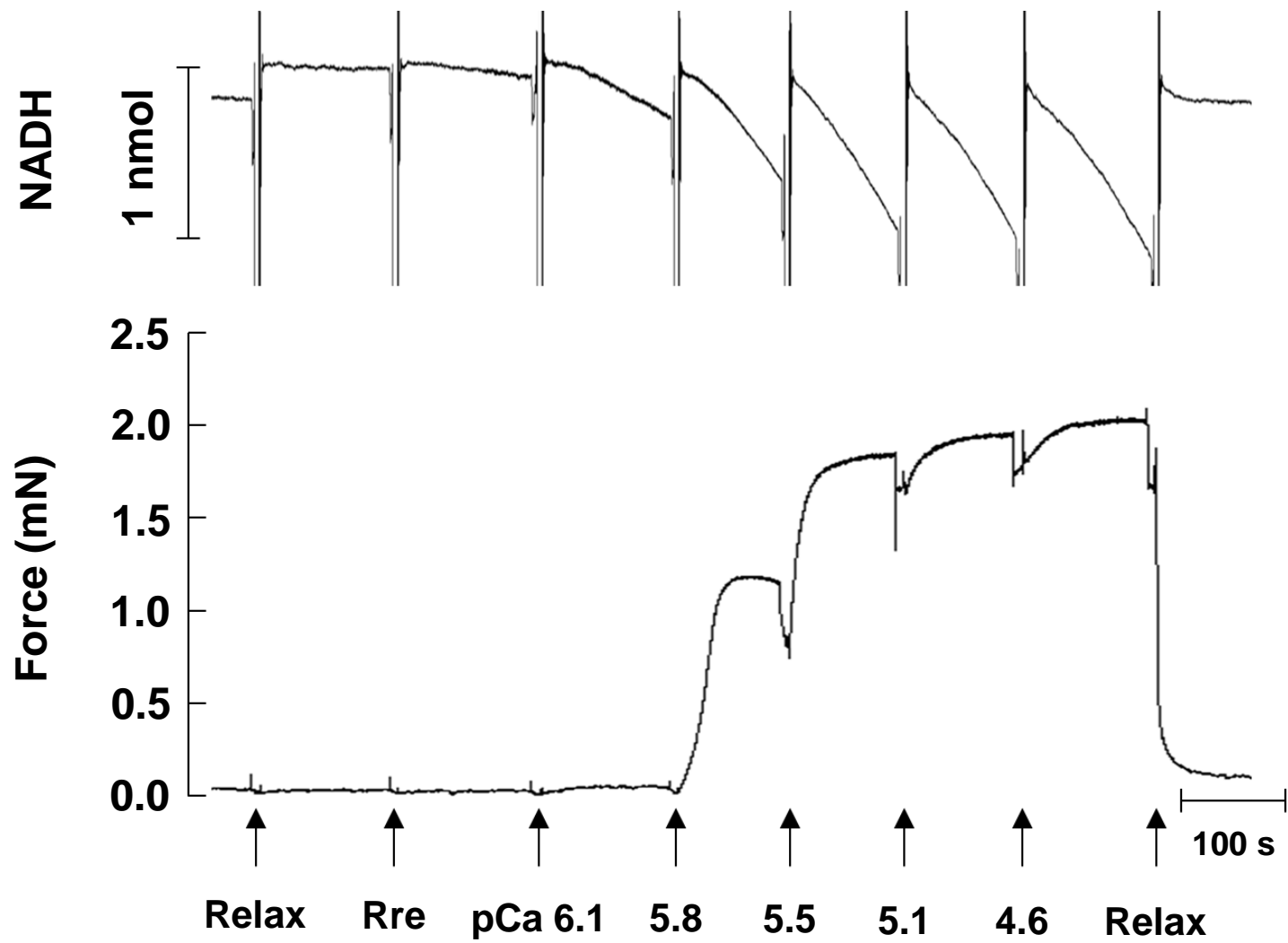

Supplement: S6 Fig — (PDF) [file pone.0215539.s006.pdf]

Fig S7

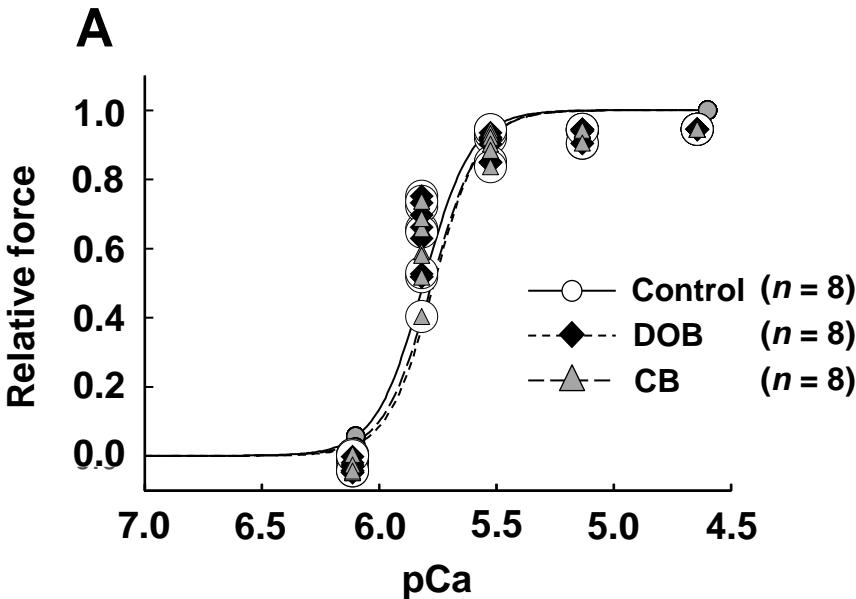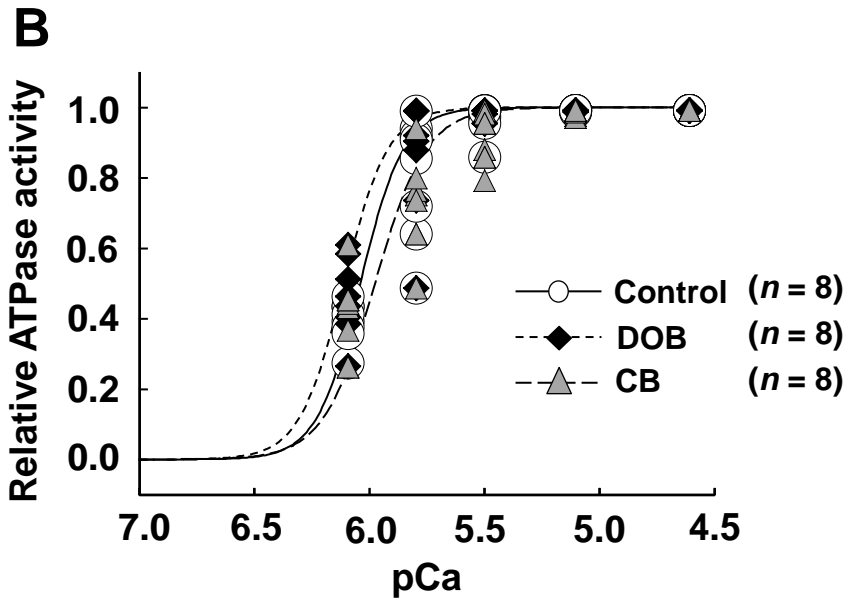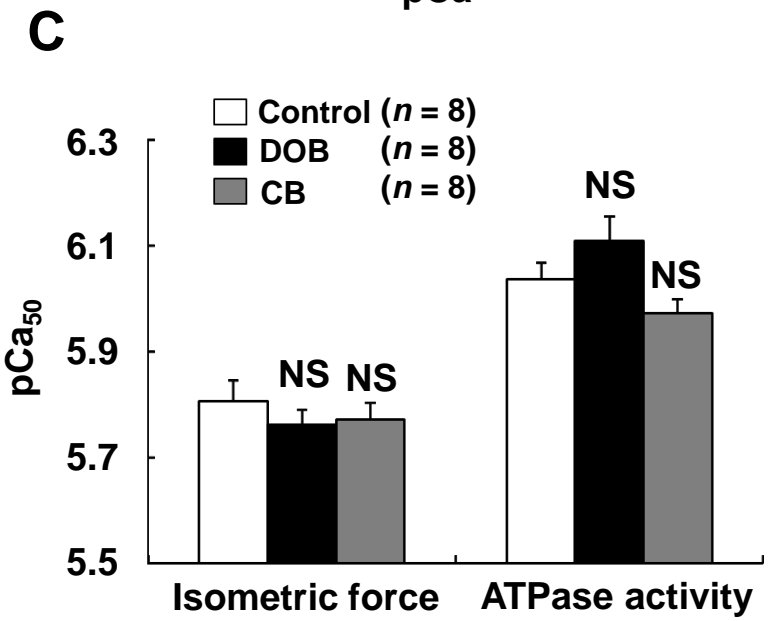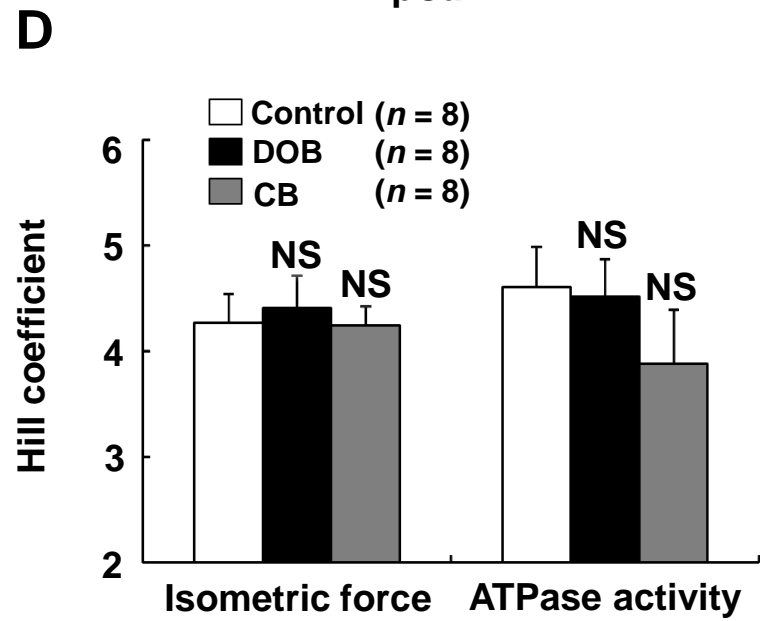

Supplement: S7 Fig — (PDF) [file pone.0215539.s007.pdf]
