# Supplementary material for: Correction: Role of β-adrenergic signaling in masseter muscle
Source: PLoS One. 2019 Jul 16;14(7):e0216752. doi: 10.1371/journal.pone.0216752 (PMC6634787; doi:10.1371/journal.pone.0216752)
Supplement: S1 Supporting information figure legends — (PDF) [file pone.0216752.s001.pdf]

Supplemental figure legends

Fig S1

Daily consumption of food and water, body weight and muscle mass.

(A-B) No significant difference in daily consumption of food **(A)** or water **(B)** was observed among the Control, DOB and CB groups (P = NS vs. Control).

(C) No significant difference in body weight (BW:g) of the Control, DOB and CB groups was observed after the 1 week treatment period.

(D) Cardiac muscle mass was significantly increased in the DOB group ($^{**}P < 0.01$ vs. Control) and the CB group ($^{*}P < 0.05$ vs. Control).

(E) Masseter muscle mass was significantly increased in the CB group ($^{*}P < 0.01$ vs. Control), but not in the DOB group.

Fig S2

Effects of DOB or CB on muscles of lower limb and TUNEL-stained sections with immunohistochemistry.

(A) Representative images of HE-stained sections of tibialis anterior muscle (fast-twitch; TA) in the Control (*upper left*), DOB (*upper right*), and CB (*lower left*) groups. The area of fibrosis was greater in the DOB group than in the CB group. The TA mass per tibial length (TL) ratio (*lower right*) was significantly increased in the CB group ($^{*}P < 0.01$ vs. Control), but not in the DOB group (P = NS vs. Control).

(B) Representative images of HE-stained sections of soleus muscle (slow twitch; SOL) in the Control (*upper left*), DOB (*upper right*), and CB (*lower left*) groups. No abnormal organization, such as fibrosis and muscle rupture, was observed, and the SOL mass per TL ratio (*lower right*) was similar among the three groups. (P = NS vs. Control).

(C) An example of images from the confocal microscopic analysis showing nuclear fragmentation of a masseter muscle (MA) myocyte in DOB group. TUNEL staining (green; *upper right*) with immunohistochemistry for simultaneous detection of dystrophin (red; *lower left)* for muscle membrane identification and Hoechst (blue; *upper left*) for visualization of nuclei confirmed that apoptotic cells identified and counted as TUNEL- positive nuclei were located inside the myofibers.

Fig S3

Effects of DOB or CB on β_1- and β_2-AR expression in differentiated C2C12 cells.

(A) Representative images of β_1- and β_2-AR expression before (D0) and at 1-6 days (D1-D6) after switch to DMEM supplemented with 2% horse serum. Both β_1- and β_2-AR expressions reached maximum at 5 days (D5) after the induction of cell differentiation.

(B) Cleaved caspase-8 expression was significantly increased after treatment with DOB (10^{-4} M) for 24 hr in differentiated C2C12 cells ($P < 0.05$ vs. Control), but not after the treatment with CB (10^{-4} M) for 24 hr.

Fig S4

Effects of DOB or CB on apoptosis in differentiated C2C12 cells.

(A-C) Representative images of triple staining for Hoechst (blue; *upper left*), PI (red; *lower left*) and myosin-heavy chain (green; *upper right*) in the Control **(A)**, DOB **(B)** and CB **(C)** groups.

(D) The number of PI-positive cells, which were also stained with an anti-myosin heavy chain antibody (white arrows), was significantly increased by the DOB treatment (P <

0.01 vs. Control), but not by the CB treatment (*P* = NS vs. Control).

Fig S5

Effects of DOB or CB on the number of microvessels per masseter myocyte.

(A-C) Representative images of triple staining for Hoechst (blue), dystrophin (red) and CD31 (green) of Control **(A)**, DOB **(B)** and CB **(C)** groups.

(D) There was no difference in the number of microvessels between the Control and CB groups (*P* = NS vs. Control). However, the number was significantly decreased in the DOB group (*P* < 0.01 vs. Control).

Fig S6

Simultaneous recording of Ca^{2+}-activated isometric force and NADH concentration in skinned masseter muscle preparation.

Simultaneous recordings of Ca^{2+}-activated isometric force (*lower*) and NADH concentration (*upper*) in a skinned masseter preparation from the control mice. The ATPase activity (rate of ATP hydrolysis) was estimated from the slope representing the rate of decrease of NADH concentration. To determine the pCa-isometric force and pCa-ATPase activity relations, skinned preparations were sequentially bathed in solutions with pCa values ranging from 8.0 (Relax or Pre) to 4.6.

Fig S7

Effects of DOB or CB on Ca^{2+} sensitivity of force and ATPase activity.

(A-B) Average (mean ± SEM) values (relative to the maximum value) of isometric force **(A)** and ATPase activity **(B)** at pCa 6.1, 5.8, 5.5, 5.1 and 4.6 were plotted for the

Control (*n* = 8, open circle), DOB (*n* = 8, closed diamond) and CB (*n* = 8, gray triangle) groups, and the data were fitted to the Hill equation (solid line, Control; dashed line DOB; long dashed line CB).

(C) Average pCa_{50} values (Ca^{2+} concentration required for half-maximal effect) of isometric force (*left*) were decreased and ATPase activity (*right*) were increased in the DOB group, but not in the CB group. However, none of the changes was statistically significant.

(D) There were no significant differences of average Hill coefficient of isometric force (*left*) and ATPase activity (*right*) among the three groups.
